# Synthesis and Characterization of Size- and Charge-Tunable Silver Nanoparticles for Selective Anticancer and Antibacterial Treatment

**DOI:** 10.1021/acsami.2c01100

**Published:** 2022-03-28

**Authors:** Barbara Pucelik, Adam Sułek, Mariusz Borkowski, Agata Barzowska, Marcin Kobielusz, Janusz M. Dąbrowski

**Affiliations:** † Małopolska Centre of Biotechnology, Jagiellonian University, 30-387 Kraków, Poland; ‡ Faculty of Chemistry, Jagiellonian University, 30-387 Kraków, Poland; § Jerzy Haber Institute of Catalysis and Surface Chemistry Polish Academy of Sciences, 30-239 Kraków, Poland

**Keywords:** advanced cellular models, antibacterial activity, anticancer activity, silver nanoparticles, organoids

## Abstract

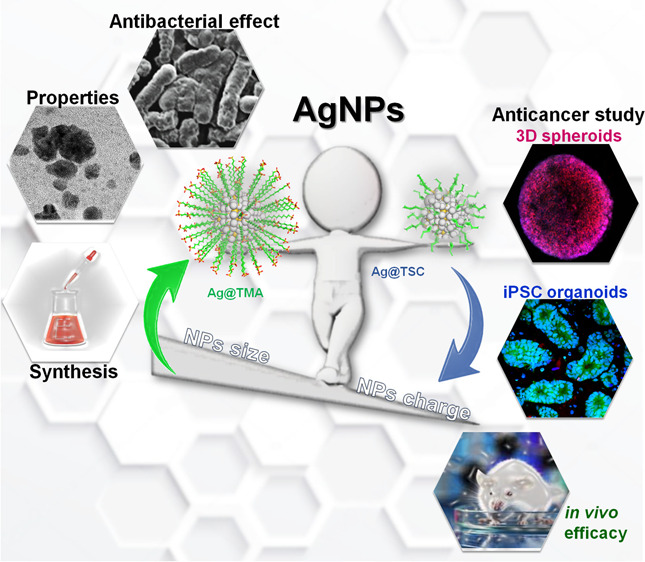

Advances in the research of nanoparticles (NPs) with controlled charge and size are driven by their potential application in the development of novel technologies and innovative therapeutics. This work reports the synthesis, characterization, and comprehensive biological evaluation of AgNPs functionalized by *N*,*N*,*N*-trimethyl-(11-mercaptoundecyl) ammonium chloride (TMA) and trisodium citrate (TSC). The prepared AgNPs were well characterized in terms of their morphological, spectroscopic and functional properties and biological activities. The implementation of several complementary techniques allowed not only the estimation of the average particle size (from 3 to 40 nm depending on the synthesis procedure used) but also the confirmation of the crystalline nature of the NPs and their round shape. To prove the usefulness of these materials in biological systems, cellular uptake and cytotoxicity in microbial and mammalian cells were determined. Positively charged 10 nm Ag@TMA2 revealed antimicrobial activity against Gram-negative bacteria with a minimum inhibitory concentration (MIC) value of 0.17 μg/mL and complete eradication of *Escherichia coli* (7 logs) for Ag@TMA2 at a concentration of 0.50 μg/mL, whereas negatively charged 10 nm Ag@TSC1 was effective against Gram-positive bacteria (MIC = 0.05 μg/mL), leading to inactivation of *Staphylococcus aureus* at relatively low concentrations. In addition, the largest 40 nm Ag@TSC2 was shown to exhibit pronounced anticancer activity against murine colon carcinoma (CT26) and murine mammary gland carcinoma (4T1) cells cultured as 2D and 3D tumor models and reduced toxicity against human HaCaT keratinocytes. Among the possible mechanisms of AgNPs are their ability to generate reactive oxygen species, which was further evaluated *in vitro* and correlates well with cellular accumulation and overall activity of AgNPs. Furthermore, we confirmed the anticancer efficacy of the most potent Ag@TSC2 in hiPSC-derived colonic organoids and demonstrated that the NPs are biocompatible and applicable *in vivo*. A pilot study in BALB/c mice evidenced that the treatment with Ag@TSC2 resulted in temporary (>60 days) remission of CT26 tumors.

## Introduction

Systemic toxicities and multidrug resistance (MDR) related to the progression and invasiveness of diseases impossible to cure completely remain major challenges in modern medicine. This is particularly relevant for MDR cancers and microbial infections, the leading cause of increasing mortality worldwide. Despite huge advances in the treatment of both cancer and bacterial infections with the use of modern technology, survival rates have remained relatively low.^1−3^ Toxic side effects of chemotherapy and surgery limit therapeutic benefit. Resistant cancers are characterized by low sensitivity to commonly used chemotherapeutics, high metastatic potential, and resistance to traditional drugs and therapeutic schemes. These factors unfavorably affect the overall prognosis of patients. Therefore, it is necessary to develop alternative and more effective anticancer strategies, including new chemotherapeutic technologies and theranostic tools, which have become a great challenge in clinical practice.^4−8^ Additionally, apart from the rapid increase of cancers displaying MDR, the drastically increasing number of antibiotic-resistant strains of bacteria leads to the search for therapeutic approaches to which microorganisms have not yet developed resistance.^9−12^ Therefore, a simple, low-cost, and high-performance multidirectional medical technology is needed to fight cancer and bacterial infections.^13−15^ It seems almost certain that nanomedicine can play a key role in overcoming existing limitations in both aspects.^16^

Nanomaterials, particularly nanoparticles (NPs), have been increasingly used in medicine due to their unique physicochemical properties, stability, as well as thermal and photochemical features.^17−19^ Among the numerous noble metal NPs, silver nanoparticles (AgNPs) have gained particular interest due to their ability to overcome the MDR problem.^20−22^ The activity of NPs is mostly derived from their ability to reach the molecular target within which specific biochemical reactions are initiated. Thus, smartly designed NPs with unique physicochemical properties thanks to their small size (<100 nm), large surface-to-volume ratio, and enhanced reactivity are excellent candidates for anticancer and/or antimicrobial agents.^23−27^

Silver salts have been widely used for various medical purposes, e.g., as antiseptic, antimicrobial, and wound healing agents, since ancient times.^28,29^ Currently, silver nitrate and AgNPs are FDA-approved for the use in antibacterial wound dressings, food supplements, and medical devices (i.e., antibacterial surface impregnation).^30,31^ Overall, AgNPs are less toxic than other forms of silver, exhibit negligible toxicity, and possess a diverse range of biomedical applications. The increasing use of AgNPs raises hopes for their use as alternative anticancer agents that may be involved in interfering with the mitochondrial respiratory chain, leading to increased reactive oxygen species (ROS) formation and ATP synthesis arrest, and consequently, DNA damage.^32−34^ So far, the significant biological activity of AgNPs against breast (MCF-7, SKBR3, and 8701-BC), leukemia, and colon (HT-29, HCT116, and Caco-2) cancer cells was demonstrated.^32−35^ AgNPs have also been well explored as vesicle-like nanosystems. For instance, AgNPs-based hydrogels embedded in polymer cross-linked networks, which were conjugated with doxorubicin have been tested as an antitumor agent against malignant melanoma. Moreover, similar hybrid nanocomposites exhibited antimicrobial activity against Gram-positive and Gram-negative bacteria.^36−38^ It was demonstrated that hydrogel containing Ag/Ag@AgCl/ZnO promotes wound healing and shows high antimicrobial activity against *Escherichia coli* and *Staphylococcus aureus* after exposure to visible light.^39^ Furthermore, Au/Ag hybrid NPs have been found to be effective theranostic agents for photoacoustic imaging and therapeutics of bacterial infections. Au-AgNPs have been used to visualize bacteria through fluorescence change analysis, making the antibacterial treatment procedure more clear and allowing accurate control of antimicrobial agent doses, thereby avoiding possible drug resistance.^40^ The toxicity of AgNPs may be modulated by their charge and size-switchable properties. For instance, zwitterionic-modified AgNPs have been shown to be highly effective antibacterial materials capable of eradicating bacterial biofilms without damaging healthy cells, indicating their potential future use in humans.^41^ AgNPs have also recently shown promise as novel virucides. AgNPs-based oseltamivir (TamifluR, neuraminidase inhibitor) carrier inhibited H1N1 influenza virus activity *via* ROS-mediated signaling pathways.^42^ Moreover, it has been shown that AgNPs can associate with coronaviruses and bind to host cells.^37,38,43^ Recently, the increasing number of virological studies have given some hope for the therapeutic use of AgNPs against SARS-CoV-2.^43,44^

In the present study, an attempt has been made to examine the effect of NP size and charge, indisputably key factors that determine the properties of NPs in biological applications. To investigate this issue, a seed-mediated growth method was used to fabricate *N*,*N*,*N*-trimethyl-(11-mercaptoundecyl) ammonium chloride (TMA)- and trisodium citrate (TSC)-capped AgNPs with three distinct size ranges optimal for biomedical applications (∼5, ∼10, and ∼40 nm). Our main goal was to obtain a range of particles with both smaller and much larger sizes (a similar approach for zeta potential), which was accomplished and demonstrated that the biological properties of each NPs fraction are fundamentally different.

**Scheme 1:**
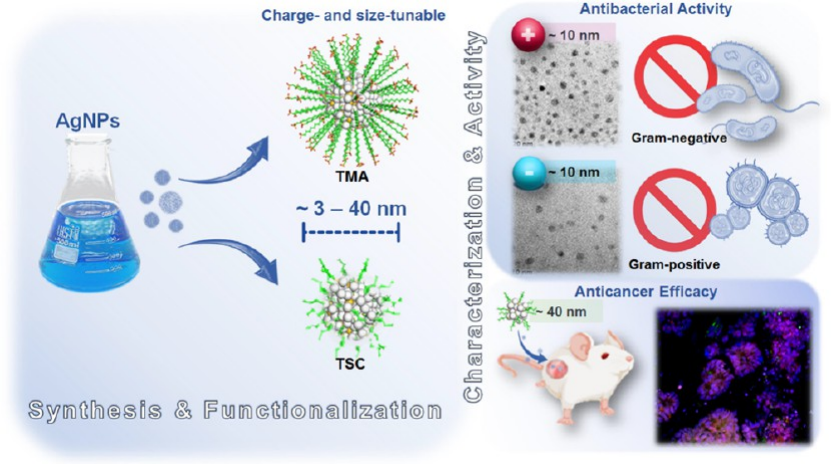
Schematic Illustration of AgNPs Synthesis, Characterization, and Biological Activity

The size- and charge-dependent activity of AgNPs against various biological models and their targeted cytotoxic properties were evaluated, starting from bacteria, through 2D and 3D cancer cell cultures, to *in vivo* efficacy (Scheme 1). Additionally, novel colonic organoid models have been used to support the recreation and adaptation of *in vitro* architectures to generate mini-organoid-like properties and their potential application to evaluate the anticancer activity caused by AgNPs.

## Results and Discussion

### Synthesis of AgNPs

**Figure 1:**
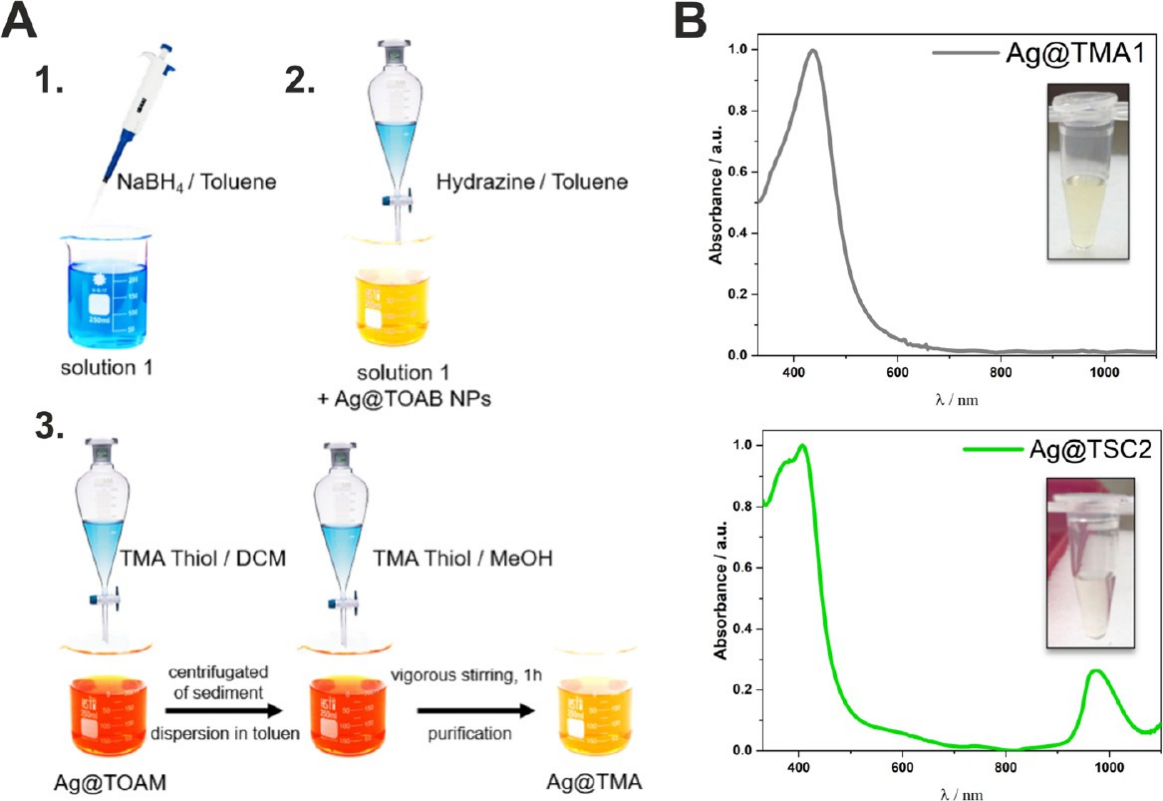
(A) Schematic illustration of the Ag@TMA and Ag@TSC NPs preparation procedures: synthesis, growth stage, and ligand exchange. (B) Electronic absorption spectra of Ag@TMA1 and Ag@TSC2 were recorded in water at room temperature (RT) and the corresponding photographs of AgNPs solution (inset).

We report a facile synthesis of multifunctional AgNPs modified by TMA, and TSC indicated as Ag@TMA and Ag@TSC, respectively. The proposed method of synthesis facilitates rapid nucleation accompanied by the growth of AgNPs at the same rate, leading to the formation of relatively monodispersed NPs. The optimum pH of the reactant medium promotes the fine-tuning of the NPs to a near-spherical shape morphology. The borohydride-mediated reduction was employed to synthesize dispersible AgNPs.^45^ An excess of sodium tetrahydroborate (NaBH_4_) was applied to produce small-sized NPs, which supported the immediate formation of nuclei, resulting in colloidal AgNPs below 10 nm in size with high levels of monodispersity and stability. The synthesis was carried out with the growth step, obtaining NPs of various sizes with a different positive surface charge. A suspension with a smaller NP diameter labeled Ag@TMA1 and a larger diameter labeled Ag@TMA2 was obtained, respectively. The process of synthesis, growing, and ligand exchange of AgNPs by direct *in situ* reduction using borohydride-mediated and TMA as a capping agent is schematically depicted in Figure 1.

The use of a weaker reducing agent, such as TSC, and a capping agent was intended to facilitate the formation of relatively larger AgNPs with a more wide size distribution.^46^ AgNPs coated with a citric ligand were synthesized according to the modified procedure described in the literature.^47^ Using an eco-friendly single-pot aqueous method, two types of NPs suspensions with different sizes and negative surface charges were obtained. The first and second wash liquors showed absorption maxima like those of the parent solution. The broad Ag@TMA absorption band at 400 nm suggests the possible presence of a population of anisotropic Ag particles (Figure 1B).

### Characterization

**Figure 2:**
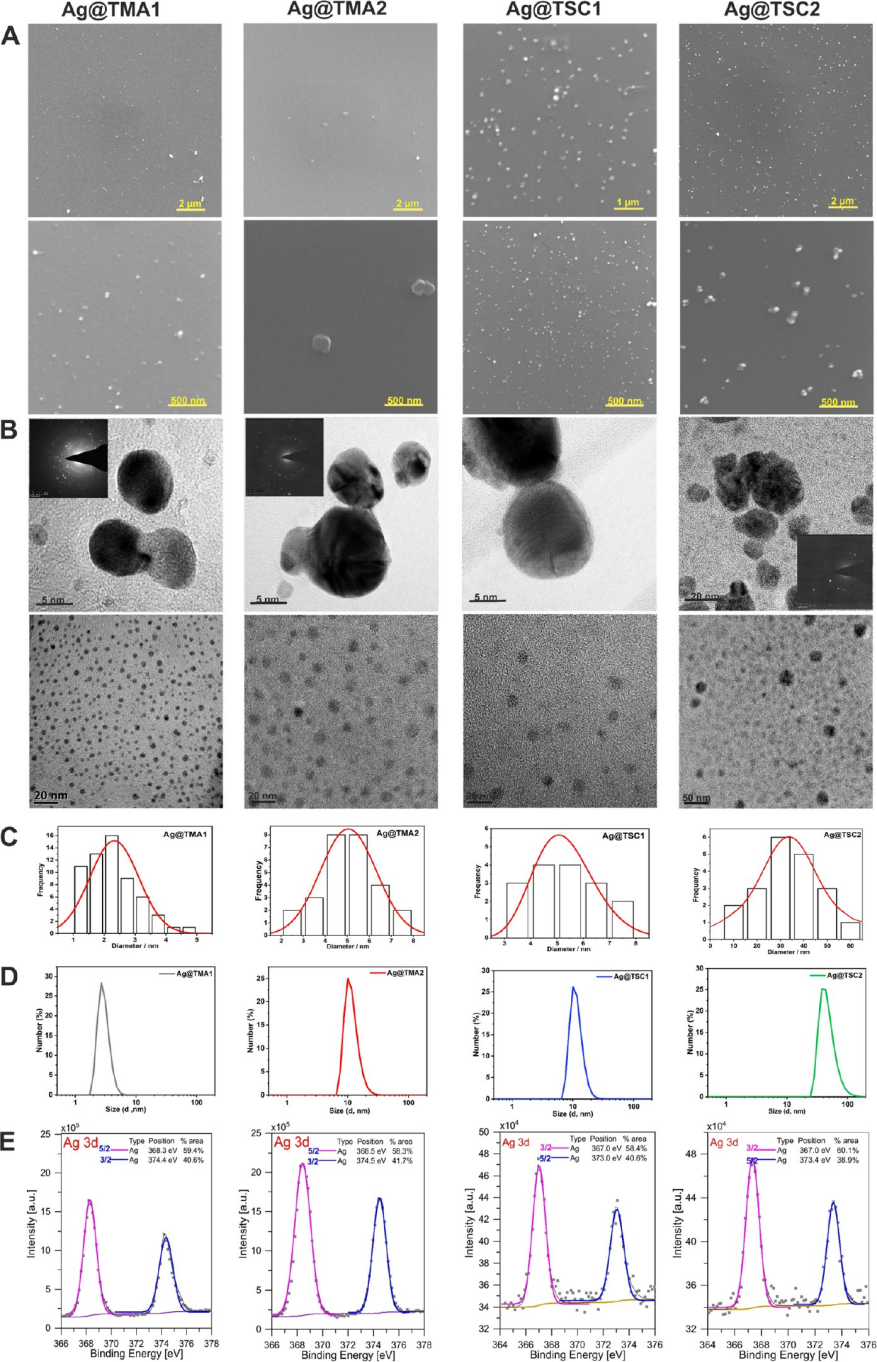
Characterization of AgNPs by detailed SEM imaging at 20 k and 100 k magnification (A), TEM images with SAED insets (B), with their corresponding bimodal size distribution (C), DLS measurements results (D), and XPS spectra (E).

The synthesized NPs possess the electronic absorption spectra typical for AgNPs with the intense band at 400 nm (Figures 1 and S1). NPs modified with TSC ligands are characterized by a relatively narrow band at λ_max_ = 400 nm and an additional band at λ_max_ = 1000 nm. NPs modified with TMA are characterized by absorption only at λ_max_ = 400 nm. Scanning electron microscopy (SEM) imaging showed that the AgNPs formed homogeneously dispersed spheres (Figure 2). The zeta potential (ξ) measurements confirmed the NPs stability (absolute value above 30 mV) and revealed that those modified with TMA have a positive surface charge, while those covered with TSC maintain a negative charge (Table 1). The composition and surface properties of AgNPs were investigated by high-resolution transmission electron microscopy (HRTEM) imaging. The selected area electron diffraction (SAED) confirmed the crystal structure of the synthesized material. The hydrodynamic diameter of NPs range from 3.2 ± 0.5 to 41.2 ± 5.0 nm and it is consistent with TEM images, which show that Ag@TMA1 NPs are smaller than Ag@TMA2. All compounds exhibit a polydispersity index (PdI) within 0.4, except Ag@TMA2 for which the PdI is approximately 0.9. The particle size and the PdI determined by dynamic light scattering (DLS) indicated the absence of aggregates (Table 1).

**Table 1:** AgNPs Size Distribution Obtained from DLS and TEM Measurements, along with Zeta Potential (ξ) and PdI Values

	size/nm		ξ/mV	PdI
NPs	DLS	TEM		
Ag@TMA1	3.1 ± 0.6	2.8 ± 1.0	+134.0 ± 9.0	0.04
Ag@TMA2	9.2 ± 1.2	11.0 ± 2.5	+93.5 ± 13.0	0.02
Ag@TSC1	11.5 ± 3.4	5.5 ± 2.5	–34.3 ± 9.6	0.09
Ag@TSC2	41.0 ± 13.7	10.7 ± 7.0	–54.7 ± 19.6	0.11

The X-ray photoelectron spectroscopy (XPS) data of the AgNPs shows two peaks in the range of 365–375 eV, corresponding to the Ag 3d_5/2_ and Ag 3d_3/2_ band, respectively (Figure 2E). These bands confirm the presence of metallic silver. However, their slight asymmetry may indicate the presence of other silver species. This possible additional silver species may originate from the Ag–O bond, which can be supported by the presence of oxygen 1s core-level peaks (Figures S3 and S4). However, the silver oxidation state analysis only based on the XPS results is not sufficient. Therefore, an SAED analysis for AgNPs was performed. In all analyzed SAED patterns, signals characteristic for silver crystals (Figure S5) and silver oxide were noticed. The XRD results for the suspension again confirmed the presence of the characteristic reflections for metallic silver (Figure S6). However, no typical Ag_2_O reflections were observed. The results of both measurements indicate the presence of Ag_2_O but in a relatively small amount. It is noteworthy that the creation of a thin layer of oxides over AgNPs is typical behavior and it was previously reported.^48^

### Stability, Thermostability and DNA Binding

**Figure 3:**
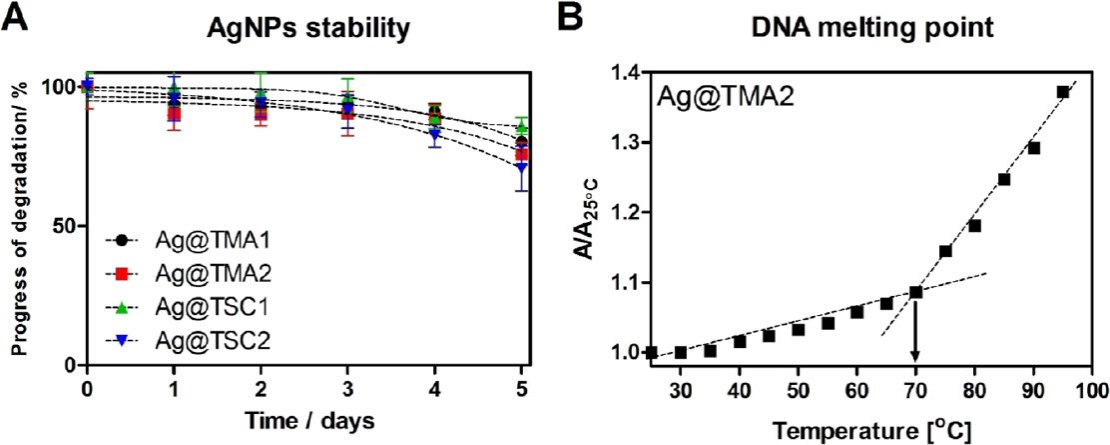
Stability of AgNPs in DMEM cell media monitored by UV–vis absorption measurements (A) and melting curves of DNA (50 mM) in the presence of Ag@TMA2 (0.50 μg/mL) (B). The results are expressed as mean ± SEM.

Regarding the biological activity of AgNPs, their two important properties were initially investigated: stability in the biological environment (Figures 3A and S7) and interaction with DNA (Figure 3B). These properties were investigated to evaluate the relative affinity to nucleic acids and, consequently, to estimate their interaction with one of the most important subcellular target of cancer and bacterial cells. Thermal stability was determined by absorption measurements over a wide temperature range (25–90°C, Figure S7). The analogous measurements recorded at different time intervals also confirm long-term stability in Dulbecco’s Modified Eagle Medium (DMEM) solution up to 5 days. Therefore, any decrease in absorption and/or increase in width at half maximum can be interpreted as AgNPs aggregating and forming microscale particles. The time-dependent disappearance of the 400 nm band suggests that the AgNPs were losing their stability as nanoscale materials. The presence of charged amino acids in DMEM increased the ionic strength and thus induced the aggregation of NPs.^49^ The changes in absorbance intensity are most probably a consequence of the interruption of the DNA double helix and are related to helix-stabilizing interactions such as H-bonds. The melting point (TM) values for ct-DNA with and without NPs were determined to be 63°C for Ag@TMA1, 70°C for Ag@TMA2, and 65°C for both citrate-coated (Ag@TSC1 and Ag@TSC2) materials (Figure S8). The TM value for ct-DNA in the absence of NPs is approximately 60°C.

### Cellular Uptake

**Figure 4:**
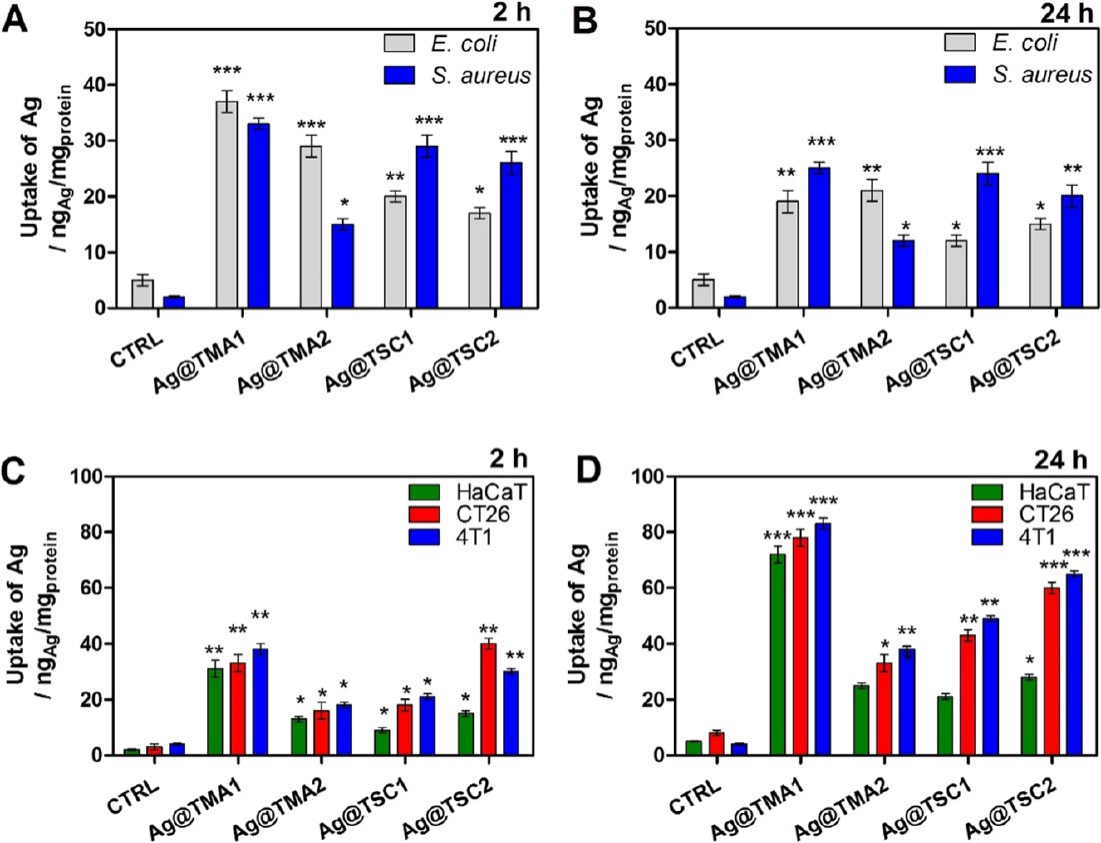
Cellular uptake of AgNPs in (A, B) microbial and (C, D) mammalian cells after 2 and 24 h incubation, determined by ICP. Data are expressed as mean ± SEM (*** *P*-value < 0.001, ** *P*-value < 0.01, and * *P*-value < 0.5).

Many research findings suggest that the antimicrobial action of AgNPs may be triggered by the disruptive interaction of functional moieties anchored by the AgNPs, which enhance their stabilization and attachment to the bacterial wall surface. Additionally, AgNPs are able to alter bacterial cells’ permeability, interfere with the cellular respiration system, and finally damage bacterial cells by reacting with proteins and DNA. Therefore, the intracellular silver concentration in cells was assessed by inductively coupled plasma (ICP) measurements after 2 and 24 h incubation of four types of AgNPs with initial concentrations of 1 μg/mL. Silver concentration in the microbial culture (*E. coli* and*S. aureus*) was already markedly increased after 2 h of incubation (Figure 4A, B). In contrast, for mammalian cells (human keratinocytes HaCaT and cancer cells CT26 and 4T1), the optimal accumulation time reaches 24 h (Figure 4C, D). The obtained results indicated that the smallest Ag@TMA1 NPs accumulate efficiently in all tested cells. This can be explained by the small diameter size (5 nm) which facilitates passive diffusion through the membranes. Moreover, it was observed that positively charged Ag@TMA2 NPs bind more efficiently to Gram-negative bacteria. This effect is also evident for citrate-coated NPs, enabling better accumulation of negatively charged Ag@TSC1 and Ag@TSC2 in Gram-positive bacteria *S. aureus*. The ultrasmall Ag@TMA1 (3–5 nm) indicated the highest cellular uptake in both species. While the uptake of positively charged TMA NPs was higher in*E. coli*, negatively charged TSC NPs accumulate more effectively in *S. aureus* than in *E. coli*, Figure 4. The observed effect may be related to the structural changes in bacterial cell wall components and appropriate fast electrostatic interaction between the NPs surface and the bacteria cell wall.^50,51^

The data from mammalian cells show that Ag@TMA1 NPs accumulate in all cell types at high Ag concentration (Figure 4B). Nevertheless, this high cellular uptake through passive diffusion may lead to higher toxicity and a lack of selectivity toward cancer cells. Since the cell membranes are mostly permeable to small and nonpolar molecules, NPs employ endocytosis pathways to enter the cells. Thus, for larger (10–40 nm) AgNPs, we noticed still significant (but lower) uptake in HaCaT, CT26, and 4T1 cells. It is widely recognized that charged molecules that are unable to pass the hydrophobic plasma membrane may undergo internalization by an active form of transport (pinocytosis).^52,53^ Thus, our data suggest that among the NPs tested, Ag@TMA2 with a diameter of 40 nm are optimal for selective accumulation in cancer cells. This is consistent with findings that NPs of 30–50 nm in size efficiently interact with membrane receptors and are subsequently internalized *via* receptor-mediated endocytosis.^52^ Moreover, the NPs uptake in cancer cells (CT26 and 4T1) is relatively higher than in HaCaT cells.

### Antibacterial Studies

**Figure 5:**
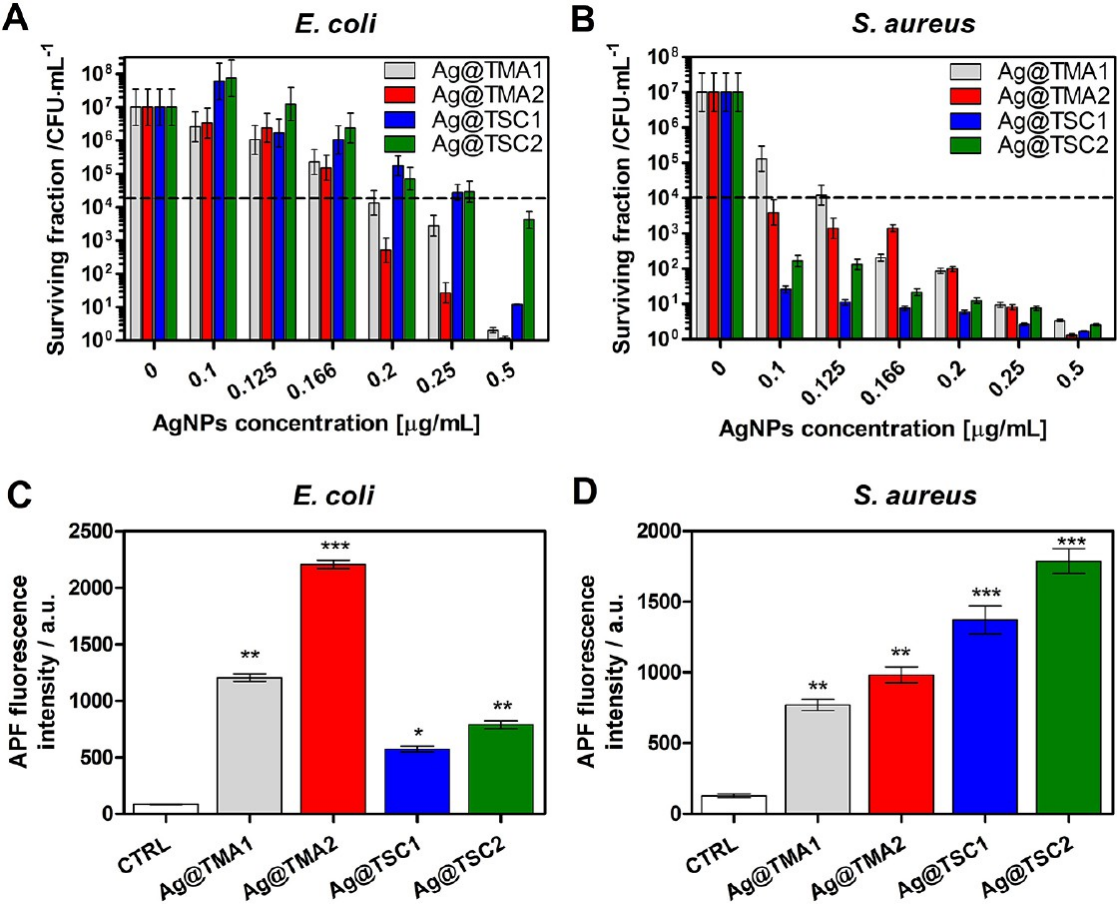
Antibacterial efficacy of investigated AgNPs against (A)*E. coli* and (B)*S. aureus* and ROS detection *in vitro* in (C)*E. coli* and (D)*S. aureus*. Data are expressed as mean ± SEM (*** *P*-value < 0.001, ** *P*-value < 0.01, and * *P*-value < 0.5).

Bacteria and other resistant microorganisms are commonly found in wounds, injured skin tissue, and immunocompromised patients. The antibacterial activity of the synthesized NP effect was tested against both Gram-positive (*S. aureus*) and Gram-negative (*E. coli*) human bacterial pathogens. The results of the bacteria treatment with AgNPs at various concentrations and 2 h of incubation are summarized in Figure 5 (Figures S9 and S10). Charged NPs interact with negatively or positively charged bacterial cell walls and lead to intracellular component leakage. As expected, negatively charged NPs (TSC) were more toxic to *E. coli* than positively charged ones (TMA). The minimum inhibitory concentration (MIC) values were calculated and are indicated in the following order: 0.20 μg/mL for Ag@TMA1, 0.17 μg/mL for Ag@TMA2, 0.30 μg/mL for Ag@TSC1, and >0.50 μg/mL for Ag@TSC2. The complete *E. coli* eradication was achieved only for Ag@TMA2 at 0.50 μg/mL concentration. For *S. aureus*, the biocidal effect was observed for all NPs, but for negatively charged NPs, the effect was more pronounced at lower NPs concentrations (from 0.10 μg/mL for TSC). The MIC values reached 0.125 μg/mL for Ag@TMA1, 0.09 μg/mL for Ag@TMA2, and 0.025 and 0.05 μg/mL for Ag@TSC1 and Ag@TSC2, respectively.

**Figure 6:**
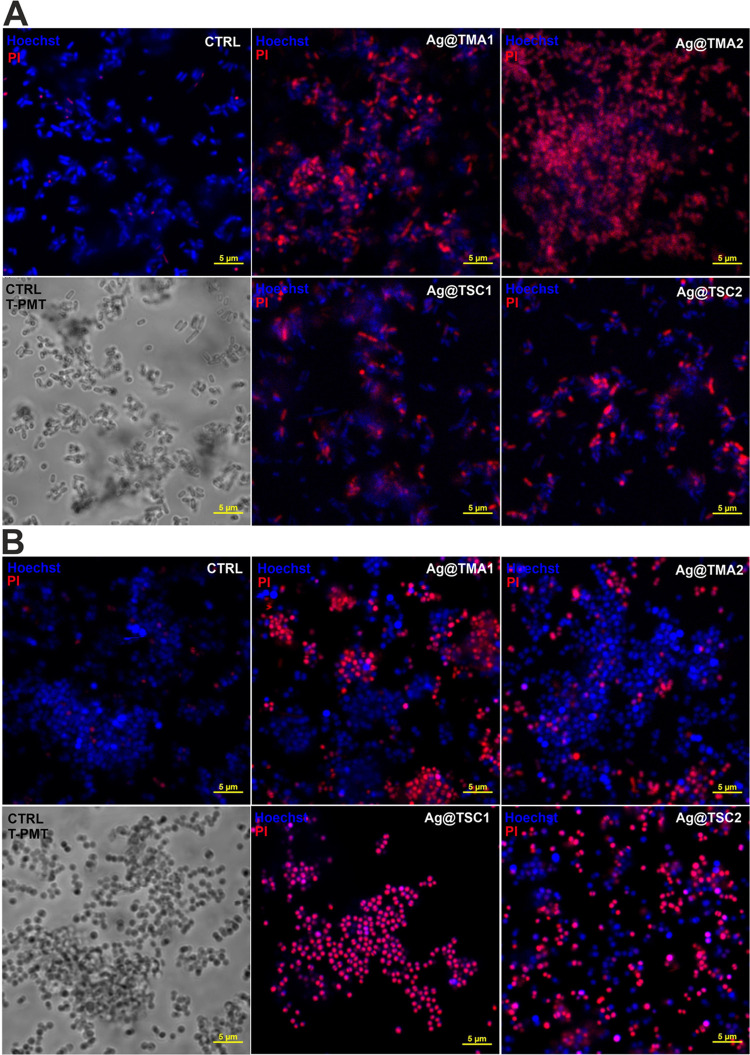
Laser scanning confocal microscopy imaging of live/dead staining of bacteria after 2 h of incubation with AgNPs: (A)*E. coli* and (B) *S. aureus*. The live cells were stained with Hoechst33342 (blue fluorescence), and dead cells were stained with propidium iodide (PI, red fluorescence). Scale bar—5 μm.

The functionalization of AgNPs by TSC and TMA leads to their higher positive/negative charge allowing interaction with cell walls and, consequently, a better antibacterial potential. Also, the NPs with a low molecular weight enable efficient penetration of bacterial cell walls and further interaction with other intracellular compartments, while high molecular weight NPs only allow the surface action. To elucidate the possible mechanism of AgNPs activity, we also determine the ability of each AgNPs to produce ROS in bacteria using a 3′-*p*-(aminophenyl)fluorescein (APF) fluorescence probe selective for oxygen-centered radicals (Figure 5C, D). As can be seen, positively charged Ag@TMA2 NPs generate ROS most efficiently in*E. coli*. For *S. aureus*, the negatively charged AgNPs are responsible for oxidative stress induction, and the highest APF fluorescence signal was observed for Ag@TSC2. ROS generation correlates well with AgNPs uptake by bacterial cells. It should also be noted that the antimicrobial activity of AgNPs may be derived from their interaction with sulfur-containing proteins in the plasma membrane that cause changes in its permeability. This may consequently lead to the leakage of intracellular components, binding to DNA and inhibiting transcription, and finally to cell death. The size and degree of dispersion of AgNPs significantly affect their antimicrobial activity. Thus, the more pronounced antibacterial effect of Ag@TMA1 may be more related to the smaller size of these NPs and, therefore, their toxicity. The smaller and better distributed the AgNPs, the more significant the bacterial inactivation. Additionally, live/dead staining and confocal imaging of AgNPs were performed to confirm the antibacterial effect (Figure 6).

**Figure 7:**
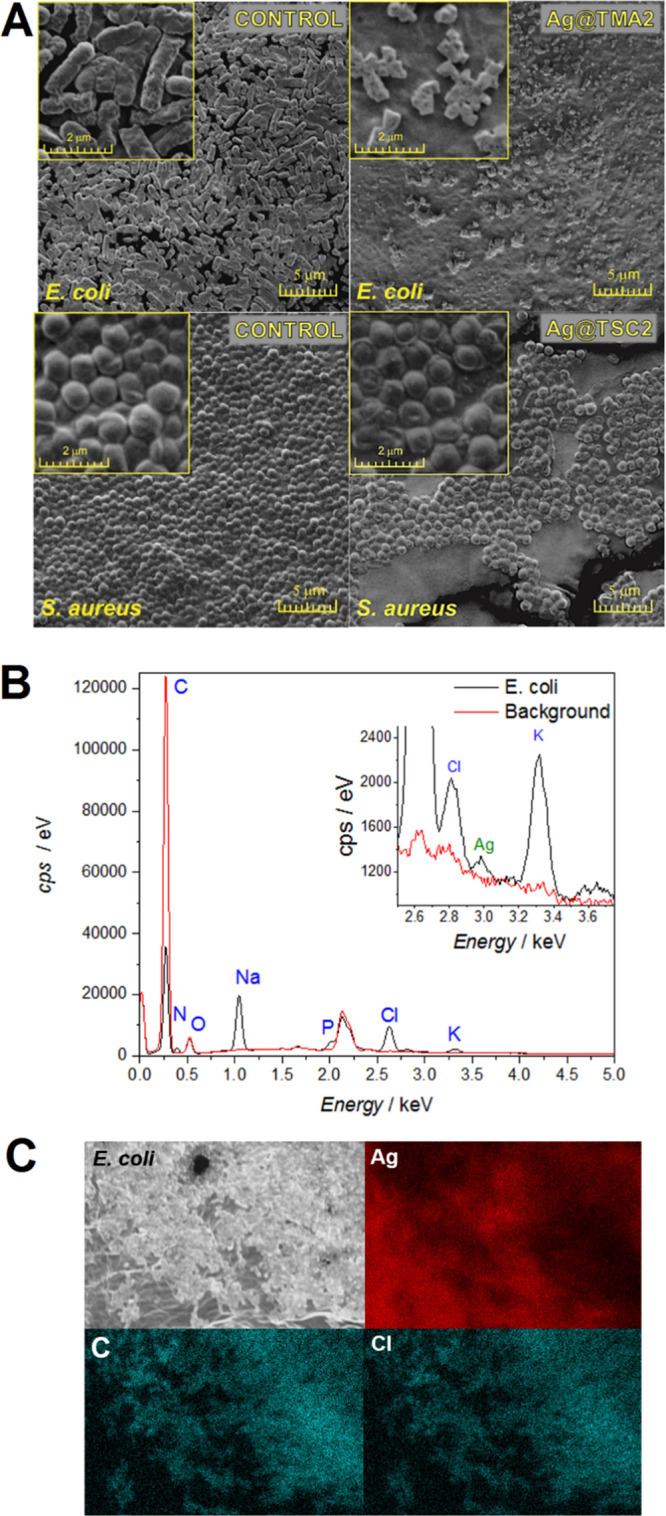
(A) Morphological changes induced by AgNPs treatment in *E. coli* and *S. aureus* determined by SEM imaging. (B) EDX spectra for the *E. coli* treated with Ag@TMA2 and (C) SEM images of Ag@TMA2-treated colonies of *E. coli* with corresponding spatially resolved EDX elemental maps: Ag, C, and Cl.

Additionally, SEM analysis was performed to evaluate the morphological changes in the bacterial cell wall upon NPs treatment. Bacterial cell structural damage induced by NPs was observed for both the tested strains (Figure 7A). The integrity of the *S. aureus* cells after exposure to Ag@TSC1 and Ag@TSC2 was severely affected by numerous perforations and cracks. Similarly, *E. coli* cells have multiple holes and strongly irregular shapes, which confirms their total disintegration. Results are in good agreement with the obtained CFU values. To verify whether the AgNPs are bound to bacterial cells, SEM EDX measurement was performed. Considering the expected small amount of silver and the relatively low sensitivity of EDX spectroscopy to elements present only on the surface, it was decided to collect the spectra from areas where bacteria are visible on the SEM image and from nearby regions without bacterial cells. The analysis showed that the signal characteristic for silver atoms is higher in the area where bacterial cells are present. Furthermore, the elemental profile of the treated bacteria analyzed using EDX revealed the presence of Ag (Figure 7B) at energy characteristics for AgNPs. It was previously reported that AgNPs showed an absorption peak of the silver element at about 3 keV.^54^ Moreover, the obtained EDX composition maps confirm a certain amount of Ag on the surface of *E. coli*, further evidencing cell/Ag@TMA2 interactions (Figure 7C), similarly to data presented by other authors.

### *In Vitro* Anticancer Activity

**Figure 8:**
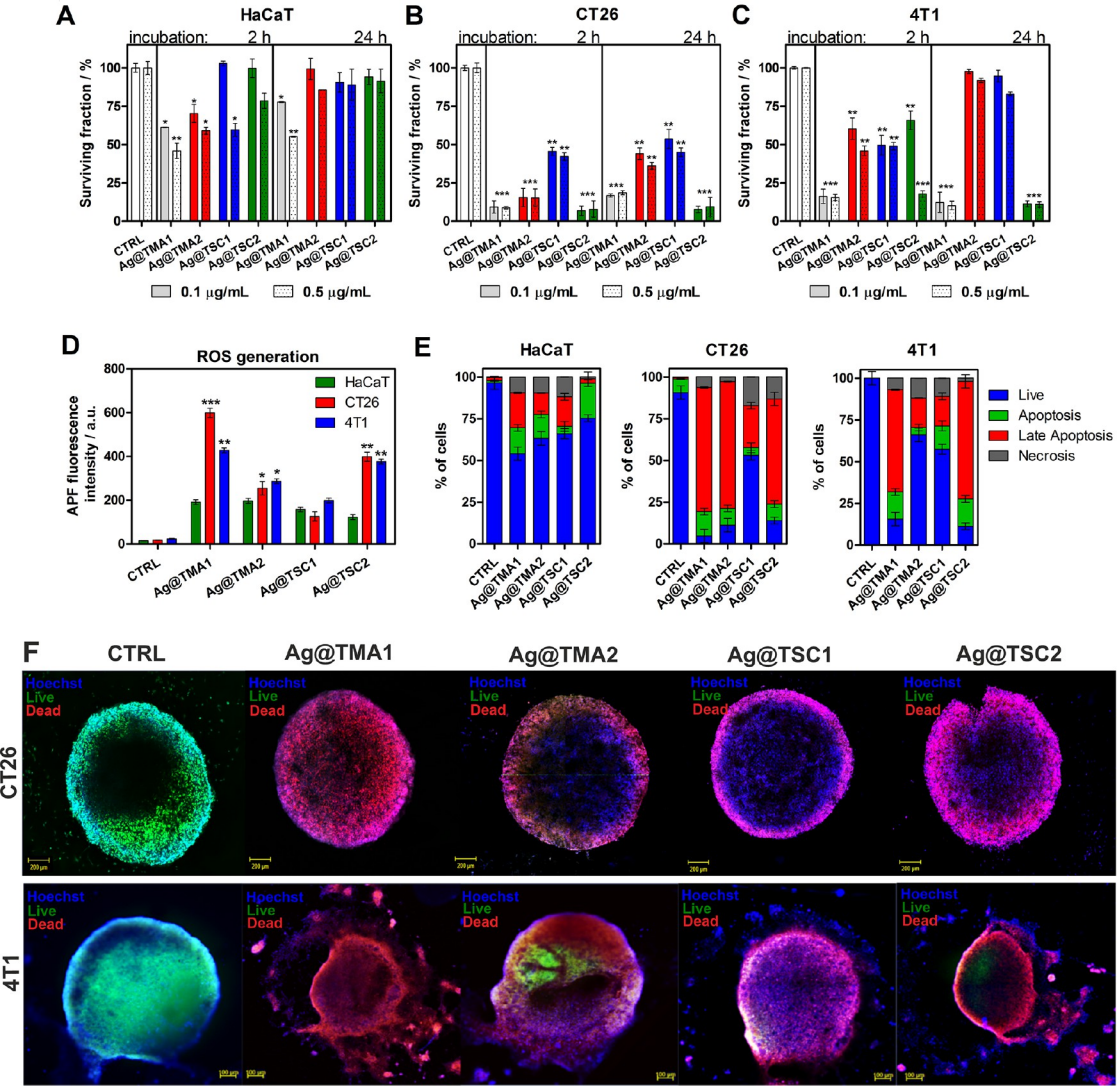
Cytotoxicity of AgNPs against (A) HaCaT, (B) CT26, and (C) 4T1 cells; *in vitro* ROS generation determined by APF fluorescence signals (D), determination of the mechanism of AgNPs-induced cell death determined by flow cytometry analysis of cells stained with Annexin V-FITC for apoptosis detection and PI for necrosis, respectively (E), and AgNPs efficacy against the 3D tumor spheroid model. Spheroids were treated with AgNPs and stained with Hoechst33342 (nuclei, blue fluorescence), Calcein AM (live cells, green fluorescence), and PI (dead cells, red fluorescence); scale bar—100 μm (F). Data are expressed as mean ± SEM (*** *P*-value < 0.001, ** *P*-value < 0.01, and * *P*-value < 0.5).

The cytotoxicity of AgNPs at concentrations of 0.50 and 0.10 μg/mL was determined by the MTT assay, which involves measuring the mitochondrial activity of cells. The MTT test was performed for noncancerous human keratinocytes (HaCaT), murine colon carcinoma (CT26), and murine mammary gland carcinoma (4T1) cells after 2 and 24 h of incubation. The results presented in Figure 8A, C show that the smallest NPs Ag@TMA1 are the most toxic to noncancerous cells (<50% mortality). Interestingly, cell viability higher than 80% (or toxicity <20%) was observed for larger (<10 nm) NPs: Ag@TMA2, Ag@TSC1, and Ag@TSC2. In contrast, AgNPs were found to be highly toxic to cancer cells. TMA1-modified AgNPs indicate high toxicity in all cases tested. The treatment of CT26 cells with Ag@TMA2 at 0.50 μg/mL results in 50–60% of dead cells and *ca.* 80% at 0.50 μg/mL, respectively. Ag@TSC1 led to the weakest effect on CT26 cells (*ca.* 50% killing), but the Ag@TSC2 caused almost complete cell death after both incubation times. In the case of more resistant 4T1 cells, a significant reduction in cell viability was observed only after treatment with Ag@TMA1 and Ag@TSC2. Notably, 10 nm-sized NPs, Ag@TMA2 and Ag@TSC1, resulted in lower toxicity at 2 h (40–50%) and 24 h (70–80%). This decrease in anticancer toxicity may be assigned to the size of NPs, affecting the kinetics of drug release to the cells and the intrinsic resistance of 4T1 cells.^55,56^ The effects of different types of investigated AgNPs suggested that especially 40 nm-sized Ag@TSC2 NPs have no significant cytotoxic effect on noncancerous HaCaT cells and are able to kill cancer cells in a targeted manner. Similar results have been described for other AgNPs.^57^ Many authors correlate the cytotoxicity and relative selectivity of AgNPs with increased apoptosis and reduced DNA synthesis in cancer cells.^58^ Thus, we assessed the ability of the tested NPs to generate ROS *in vitro* (Figure 8D) and were tempted to elucidate the cell death mechanisms (Figures 8E and S11). The obtained results indicate that the most effective Ag@TMA1 and Ag@TSC2 generate intracellular ROS more efficiently than Ag@TMA2 and Ag@TSC1. Moreover, ROS production is higher in cancer cells than in noncancerous HaCaT, which correlates with cellular uptake and NP anticancer activity. The apoptosis and necrosis of HaCaT, CT26, and 4T1 cells were evaluated by flow cytometry. The percent of live, apoptotic, late apoptotic, and necrotic cells after incubation with 0.50 μg/mL AgNPs for 24 h is shown in Figure 8E. As depicted in Figure 8E, the percent of apoptotic, late apoptotic cells, and necrotic cells was significantly higher than the control cells. In contrast to cancer cells, Ag@TMA1 induces cell death up to 50% of stained cells in HaCaT cells. For other NPs, this effect decreases, leading to *ca.* 25% apoptotic cells after Ag@TSC2 treatment. In contrast to apoptosis, we also observed a slight increase in the necrotic cell population (up to 15%). In the CT26 and 4T1 cells incubated with AgNPs, we observed a marked increase in late apoptosis, indicating that apoptotic cell death is a major mechanism involved in AgNPs activity. Other studies indicate that AgNPs can cross cell membranes and may interact with cellular components and organelles with ROS generation, leading to decreased antioxidant cell capacity, direct and/or indirect interaction with DNA, and apoptosis.^59−61^

Due to the fact that tumor cell spheroids with 200–500 μm diameter are known to evolve oxygen, nutrient, and energy gradients comparable to those found *in vivo*,^62^ we prove the AgNPs anticancer efficacy in 3D tumor spheroids established for CT26 and 4T1 cells. Confocal microscopy imaging of spheroids (Figure 8F) shows that after treatment with AgNPs, the cells in the spheroids were mostly dead as assessed by a live/dead assay using the Calcein AM dye to mark live cells and the PI to label dead cells (Figure 8F).

It can be concluded that the best NPs for anticancer studies may be Ag@TSC2 due to the observed selectivity toward cancer cells and the lowest toxicity in noncancerous cells. This effect may be attributed to cell metabolism kinetics, which is faster in cancer cells than in normal cells, thereby enhancing the intracellular release of AgNPs in cancer cells.

Our study confirms that the size of AgNPs act as a crucial factor in defining the mechanism of AgNPs interaction with the biological system. In general, the sizes of NPs are similar to those of proteins (∼2 to 10 nm), DNA helix diameter (∼2 nm), and the cell membrane thickness (∼10 nm). This means that NPs are capable of easily entering cells and targeting cell organelles. It was also shown that AuNPs >6 nm may efficiently penetrate the cell nucleus, while larger NPs (10 or 16 nm in size) only pass through the membrane and localize mainly to the cytoplasm. Thus, it can be concluded that NPs with sizes below 10 nm exhibit greater toxicity than those of 10 nm or larger because the larger NPs are unable to enter the nucleus.^63^ Similarly, the size-dependent toxicity of AuNPs has been demonstrated. NPs with 15 nm size are significantly less toxic (*ca.* 60 times) for various types of cells than NPs with 1.4 nm diameter.^64^ These data indicate not only that NPs can enter the nucleus, but the ultrasmall NPs can also interact with the negatively charged DNA backbone and block transcription because their size is consistent with that of the DNA major groove. Furthermore, ultrasmall NPs particles (<5 nm in size) typically cross cellular barriers in a nonspecific manner, such as through translocation. On the other hand, larger NPs enter cells by bulk transport processes such as phagocytosis, pinocytosis, or specific/nonspecific transport mechanisms. In general, NPs with a diameter of 10–100 nm may be considered as suitable for anticancer treatment due to effective delivery and enhanced permeability and retention (EPR) effects. Smaller NPs may undergo rapid release from the normal vessels and damage healthy cells and tissue prior to their metabolism by the kidneys (<10 nm in size).^65,66^ Accordingly, we also observed the highest toxicity for Ag@TMA1 (<5 nm in size). Moreover, the activity of Ag@TMA1 is less selective and can destroy each type of cells, which limits their application in targeted treatment. Therefore, based on our data, it can be concluded that Ag@TSC2 are the most promising candidates for anticancer treatment due to their larger size (30–50 nm) and thus the possible EPR effect and improved selectivity toward cancer cells.

### AgNPs Cytotoxicity in hiPSC-Derived Colonic Cancer Organoids

**Figure 9:**
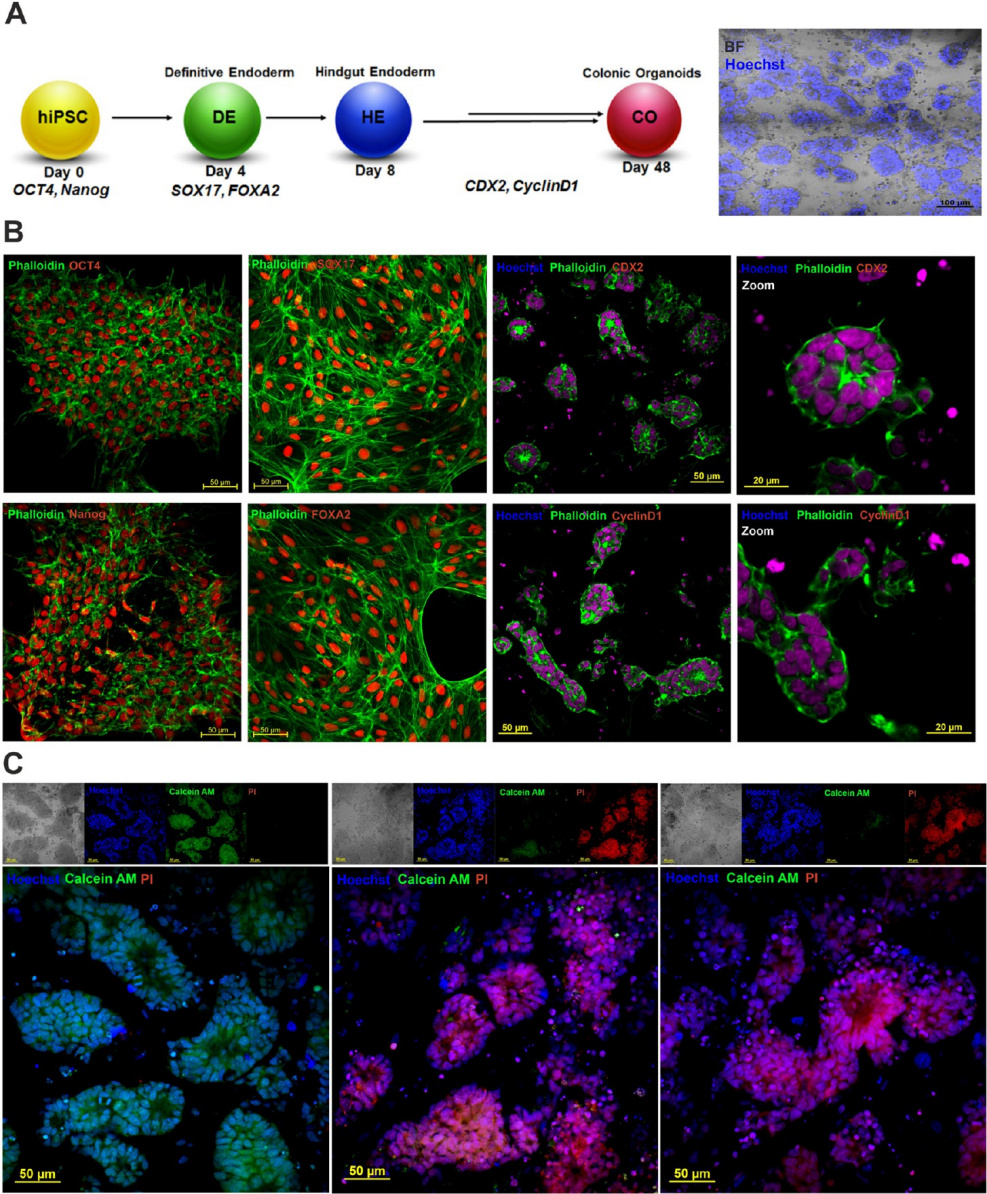
(A) Scheme illustrating the differentiation of iPSC to iPSC-derived colonic organoids, (B) pluripotency and colonic tissue markers staining: phalloidin—green, OCT-4, SOX17, Nanog, FOXA2, CDX2 and cyclin D1—red, and Hoechst 33342—blue; scale bars—50 μm, and (C) live/dead staining of colonic organoids after treatments with AgNPs: Calcein AM—green, PI—red, and Hoechst 33342—blue, scale bars—50 μm.

Organoids engineering bridges the gap between traditional 2D cell cultures and *in vivo* models.^67^ We have developed hiPSC-derived colonic organoids (also named enteroids or miniguts), which preserve the physiological features of the digestive system and are a powerful tool for studying human intestinal diseases, including colorectal cancer. First, the pluripotency of the hiPSCs was confirmed by OCT4 and Nanog staining. We then used an established procedure to generate colonic organoids for direct differentiation of hiPSCs to definitive endoderm (DE), confirmed by SOX17 and FOXA2 expression (Figure 9), followed by the formation of colonic spheroids verified by CDX2 staining (Figure 9A, B).^68^ The deregulated degradation of cyclin D1 appears to be responsible for the increased cyclin D1 levels in many cancers associated with their development and further progression (Figure 9B). The organoids were then cultured in Matrigel for 48 days. The obtained cancer colonic organoids consisted of columnar epithelium patterned with villus-like structures (Figure 9A, B).

To investigate the anticancer potential of AgNPs, we performed live/dead staining of untreated and Ag@TMA1- and Ag@TSC1-treated organoids with Hoechst 33342, Calcein AM, and PI. Confocal laser scanning microscopy (CLSM) imaging revealed that the untreated organoids were Hoechst- and Calcein AM-positive but PI-negative, indicating the high viability of these organoids. Notably, after NPs administration, most cells in the organoids indicate significant increase in PI-positive cells, demonstrating the induction of cell death. As may be seen in the bright-field images, this was accompanied by a disrupted organoid structure (Figure 9C).

It is worth mentioning that the results obtained from the organoids have a huge translational potential. It has been found that organ-specific three-dimensional cell clusters derived from hiPSC or cancer-like organoids are organized in the same manner as cell sorting and spatial restriction and distribution of cells *in vivo*, making them an ideal model for mimicking human cancer features and cancer cell heterogeneity.^69^ Colonic organoids derived from iPSC have been found to be a promising alternative because they avoid the need for primary resections, which are often rare.^68^

### AgNPs Anticancer Activity *In Vivo*

**Figure 10:**
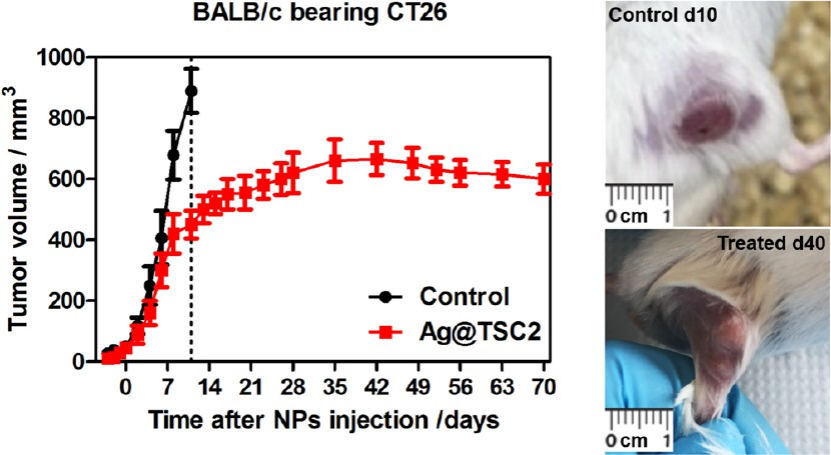
*In vivo* efficacy of Ag@TSC2. The kinetics of CT26 tumor growth in BALB/c mice after injection of Ag@TSC2 (left) and photographs of representative tumors from the control and treated group.

Based on the *in vitro* activity of investigated AgNPs, the antitumor efficacy *in vivo* was performed for the most promising Ag@TMA2 using the subcutaneous CT26 tumor-bearing mice model. For this purpose, BALB/c mice bearing CT26 tumor received intraperitoneal (*i.p.*) injections of Ag@TSC2 or vehicle (control group) twice a week after the inoculation of CT26 cells. As indicated in Figure 10, the mean tumor size was similar in each group at the beginning of the experiment. The initial tumor volume 7 days after inoculation is indicated as a day “0” in Figure 10. At this time point (start of treatment), the tumor volume reaches *ca.* 60 mm^3^ for the control and the AgNPs-treated groups, respectively. It can be observed that after the administration of AgNPs, the initial tumor size was significantly smaller in the AgNPs-treated group compared to the control group. In control animals, tumors grew faster, and mice were sacrificed when they reached more than 1 cm in diameter (*ca.* 900 mm^3^). AgNPs treatment caused significantly slower tumor development than that of the control group. The applied AgNPs treatment prevents tumor growth, suggesting that AgNPs exhibited significant anticancer effects against CT26 tumors *in vivo*.

## Conclusions

We successfully prepared and characterized smartly engineered AgNPs capped with TMA and TSC for nanomedical applications. Using a low-cost and eco-friendly sodium borohydride-based procedure, it was possible to achieve homogeneous growth of nanometric grains of AgNPs through tailoring and optimizing reaction parameters and ligand concentration. We used this method to obtain AgNPs with precise control of their size in the range from 3 to 40 nm. We applied versatile physicochemical methods (*i.e.*, DLS, XPS, SEM, EDX, and TEM) to characterize investigated AgNPs in detail, as well as cell biology techniques allowing the study of the size- and charge-dependent biological properties toward bacteria (*E. coli* and *S. aureus*) and cancer cells using *in vitro* (4T1 and CT26 cells cultured as monolayers and 3D spheroids, stem cell-derived colonic cancer organoids) and *in vivo* models (BALB/c mice bearing CT26 tumor). The synthesized NPs retain excellent stability in water and biological media. Moreover, these size- and charge-tunable AgNPs show unprecedented performance as antimicrobial and anticancer agents. The observed AgNPs-induced cytotoxicity may be attributed to efficient cellular uptake of NPs, which induces oxidative stress. ROS generation and the cellular redox state impairment allow the NPs to easily cross the cell membranes and interact with DNA, resulting in apoptosis and ultimately cell death. Furthermore, Ag@TSC2 treatment of mice bearing colon carcinoma (CT26) significantly decreased tumor growth kinetics compared to nontreated control animals, making these AgNPs worth further investigation, especially *in vivo*. Our findings suggest that these AgNPs with multiple, targeted potential activities could be greatly utilized in managing various diseases, including cancer and MDR bacteria. In addition, the obtained results highlight the significant potential of organoid–NPs interaction for the development of advanced therapeutics and design of nanomedicine-based treatments using organoid technology as an alternative cellular model to traditional cell-based drug toxicity testing to further validate the anticancer potential of AgNPs.

## Materials and Methods

### Chemicals

TSC, silver nitrate (AgNO_3_), tannic acid (TA), oleyamine (OA), tetra-*N*-octylammonium bromide (TOAB), NaBH_4_, hydrazine monohydrate (N_2_H_4_), TMA, polyethyleneimine (PEI), and dextran sulfate salt (DSS) were purchased from Sigma-Aldrich. Dry and ultrapure toluene, dichloromethane, and methanol purchased from Sigma-Aldrich were used as solvents. Distilled water from a Millipore system (ρ = 18.2 MΩ) was in all experiments and also when washing glass parts of the laboratory equipment.

### Synthesis of Ag@TMA NPs

Ag@TMA were synthesized in the following steps: AgNO_3_ (20 mg) was fully dissolved under sonication in a toluene solution (13.2 mL), which additionally contained OA (0.41 mL) and TOAB (654.0 mg). In a separate flask, NaBH_4_ (17.0 mg) and TOAB (260.0 mg) were fully dissolved by sonication and then heated (50 °C) in toluene (5.7 mL). The NaBH_4_ mixture was injected into the solution from the first step (solution I), with vigorous stirring. The solution was stirred overnight to acquire Ag@TMA NPs dispersion. Additionally, the solution changed its color to dark brown. The seed-mediated growth process was carried out using a modified solution I (AgNO_3_, 192.0 mg; OA, 4.30 mL; and TOAB, 1.39 g dissolved in 113.4 mL of toluene) mixed with seed solution under vigorous stirring. In a separate flask, N_2_H_2_ (289.9 μL) and TOAB (1.39 g) were dissolved in toluene (50 mL), then dropped into solution I with AgNPs for 30 min, under vigorous stirring. As previously mentioned, the solution was stirred overnight. Functionalization with ligand exchange by TMA (28.0 mg) in dichloromethane (DCM; 20.0 mL) was added to a solution of Ag@TOAB NPs in toluene (50 mL). The precipitate was centrifugated at 10000 rpm and was washed several times with DCM. In the next step, TMA (14.0 mg) in MeOH (10.0 mL) was added to the precipitate. The ligand exchange process was continued under vigorous stirring over 1 h. The obtained AgNPs were precipitated by the addition of toluene (100 mL) and washed several times with a mixture of DMC and toluene to remove residual reagents. Finally, the Ag@TMA NPs precipitate was redispersed in H_2_O (1.5 mL).

### Synthesis of Ag@TSC NPs

The synthesis of Ag@TSC consist of two steps. In the first step, 20 mL of TSC solution (25 mM) was mixed with 2 mL of TA solution (5 mM) and diluted with 78 mL of water. The obtained solution was stirred for 15 min under reflux conditions, then 1 mL of AgNO_3_ solution (25 mM) was added dropwise into the solution. The change in the color of the solution, from bright yellow, through intensive yellow to slightly red, within several minutes indicated the formation of AgNPs coated with the citric ligand. After the reaction, the final solution was purified by recentrifugation at 2500 rcf, which led to the separation of NPs larger than 40 nm from the supernatant. The purification process was carried out by recentrifugation at 18,000 rcf and then the solution was washed several times, resulting in a precipitate of AgNPs with water. Finally, the acquired seeds were redispersed in 20 mL of 2 mM TSC solution. The size and zeta potential of the final AgNPs were estimated by DLS, SEM, and TEM. The previously obtained Ag@TSC NPs, without the purification step, 10 min after the synthesis, were divided into two equal parts. First, 50 mL of hot water was added into the dispersion of NPs; second, 1 mL of TSC solution and 1.5 mL of TA solution were added. The dispersion was vigorously stirred for 5 min under reflux after which 1.5 mL of AgNO_3_ solution was added dropwise. The reaction was run for additional 5 min and then the AgNPs were isolated and purified as in the previous step.

### UV–VIS–NIR Electronic Spectra Measurements

The samples of AgNPs at a concentration of 1 μg/mL were prepared in distilled water. The electronic absorption spectra were registered in quartz cuvettes (l = 1 cm). The measurements were performed at RT and the range of 350–1050 nm, using a Shimadzu 3600 UV–VIS–NIR spectrophotometer.

### Size and Charge Measurements

NPs size and charge were measured with a Zetasizer Nano ZS (Malvern Panalytical) in the configuration of a measurement angle of 173°.

### Silicon Substrate Treatment for NPs Microscopy Observations

The procedure is based on thoroughly washing the silicon substrate (1 cm × 1 cm) in methanol and then ultrapure water under sonication treatment. In the next step, ultraclean and dried under argon flush substrates were oxidized by an oxygen plasma treatment (25 mL/min flow rate of O_2_; 15 min). Polyelectrolytes (PEI or DSS) were applied onto the surface of the silicon substrate by the layer-by-layer (LBL) technique. In the case of the negative charge of NPs, one PEI layer was formed on the surface as a result of immersing substrates in a PEI solution (1 mg/mL; 30 min). In the case of the positive charge of NPs, an additional second DSS layer was formed onto the PEI layer by immersing in DSS solution (1 mg/mL, 30 min). The deposition of the NPs was carried out by immersing the appropriate substrates (for negatively charged NPs—substrates with positive PEI layers; for positively charged NPs—substrates with negative DSS layers) in the NPs’ dispersion during 20 min. Freshly prepared solutions of polyelectrolytes, which were previously sonicated, were used.

### Transmission Electron Microscopy

TEM imaging of investigated AgNPs was carried out using a high-resolution analytical transmission electron microscope (FEI Tecnai Osiris) equipped with an X-FEG Schottky field emitter (200 kV) accordingly to the protocol reported by us in ref (5).

### SEM Imaging

The AgNPs, as well as bacteria images, were obtained with SEM Tescan VEGA 3 with a LaB_6_ emitter following the procedures described in our previous work.^9,70^

### X-ray Photoelectron Spectroscopy

XPS analyses were performed using experimental conditions and procedures described in our previous work.^70^ The binding energies of the Ag^+^, N^+^, S^2–^, and O^2–^ components in NPs were corrected for the C 1s line in the respective samples. Spectra analysis was performed with Voigt-shaped peaks and subtraction of a Shirley background using CasaXPS Software.

### Stability of AgNPs

The solutions of investigated AgNPs were prepared by diluting in a standard cell culture medium, DMEM. AgNPs solution in DMEM with absorbance *ca.A* = 0.1 a.u. was kept at 37 °C to mimic biological experimental conditions for various times, and, at each time point, the electronic absorption spectra of AgNPs were recorded using a Shimadzu 3600 UV–VIS–NIR spectrophotometer. The changes of the main absorption band *ca.* 400 nm were monitored, and the decrease in its intensity was used to assess AgNPs stability.

### Thermostability of AgNPs and DNA Binding

The measurements were performed using a thermostat to obtain a stable temperature in the range of 25–90°C. To prepare ct-DNA with a concentration of 0.1 mM, a suitable amount of each nucleobase was dissolved in 0.1 M NaOH solution. One milligram of the obtained DNA was then dispersed in 2 mL of Tris–HCl buffer (0.1 M), and AgNPs were added to a final concentration of 0.50 μg/mL. Afterward, a UV–vis cuvette containing the sample was kept at thermal equilibrium for 5 min before recording the spectra at each selected temperature. When the required temperature was achieved, the electronic absorption spectra in the range 200–800 nm were recorded using a Shimadzu UV–VIS–NIR 3600 spectrometer to determine the DNA melting point.

### Inductively Coupled Plasma Mass Spectrometry (ICP-MS) Measurements

Bacteria (10^7^ CFU/mL of *E. coli* and *S. aureus*) and mammalian cells (10^6^ of HaCaT, CT26, and 4T1 cells) were treated for 2 and 24 h with 1 μg/mL of four types of AgNPs. After this incubation, the intracellular silver concentration was evaluated by inductively coupled plasma mass spectrometry (ICP-MS). For ICP analysis, cells were mineralized in 1 mL of 65% HNO_3_, and the protein content in each sample was determined using Bradford assay. Next, the silver ion concentration was determined using an ICP-MS spectrometer (ELAN 6100 PerkinElmer). The silver concentration in the analyzed samples was expressed as ng Ag/mg protein. The experiment was performed at least three times independently, and results were presented as mean ± standard deviation (SD).

### Antibacterial Studies

#### Bacterial Strains, Culture Conditions, and Toxicity of AgNPs

Bacterial cell growth conditions and methods for the investigation of drug-mediated cytotoxicity against *E. coli* and *S. aureus* were described in refs (9, 11, 70). In studies performed in this work, bacteria (*E. coli* and *S. aureus*) were treated for 2 or 24 h with various concentrations (0–0.50 μg/mL) of each AgNPs.

#### CLSM Imaging of Bacteria

The live/dead staining and confocal imaging of AgNPs was performed according to the methods and procedures described in our previous work.^9^ Bacteria were incubated with the NPs solution (0.50 μg/mL) for 2 h. After washing, the bacteria samples were counterstained with PI and Hoechst33342.

### *In Vitro* Anticancer Activity

#### Cell Culture

HaCaT (human immortalized keratinocyte cell line) and CT26 (mouse colon carcinoma, ATCC: CRL-2638) cells were grown in high-glucose DMEM (PAN Biotech). 4T1 cells (mouse, mimics human breast cancer; ATCC: CRL-2539) were cultured in RPMI-1640 (PAN Biotech). All the culture media were supplemented with 10% FBS (PAN Biotech) and 1% of antibiotics (100 IU/mL penicillin + 100 mg/mL streptomycin). The cells were kept in incubators at 37 °C and 5% CO_2_ under fully humidified conditions. All experiments were performed on cells in the logarithmic phase of growth. Media were replaced every 2 days, and cells were passaged using 0.25% trypsin–EDTA (PAN Biotech). The cell culture methods and conditions were previously reported in our previous papers.^5,6^

#### Cytotoxicity and Cell Viability Assay

To quantify the AgNPs-induced cytotoxicity, the MTT test was used, followed by the procedure described in our previous work.^5,6^

In this experiment, HaCaT, CT26, and 4T1 cells were seeded, and after cells attachment, AgNPs solution at a concentration of 0.10 and 0.50 μg/mL was added to the cultures. The treated cultures were incubated for 2 or 24 h. Next, cells were rinsed with PBS, and a fresh completed culture medium was added to each well. After 24 h, the MTT test was performed.

#### Detection of ROS *In Vitro*

An APF fluorescent probe was used for detection of ROS formation according to the method described in our previous work.^5^ In these experiments, cells were treated with 0.50 μg/mL solution of NPs prepared in DMEM medium for 24 h. Two hours prior to the end of incubation, APF at a concentration at 20 μM was added to the cells. Then, cells were rinsed two times with prewarm PBS, 100 μL of HBSS was added to each well, and fluorescence intensities were measured from treated cells and appropriate controls with the following parameters: λ_exc_ = 488 nm, λ_em_ = 515 nm using a microplate reader (Tecan Infinite M200Pro Reader).

#### Flow Cytometry Analysis and Death Mechanism Examination

Flow cytometry analysis was performed using the experimental conditions, and the overall procedures are described in our previous work.^6^ HaCaT, CT26, and 4T1 cells were stained using an Annexin V/Dead Cell Apoptosis Kit (LifeScience Technologies) according to the manufacturer’s instructions. Cells were measured by a Guava flow cytometer (Merck Millipore), and the results were analyzed by InCyte software (Merck Millipore).

#### 3D Tumor Spheroid Culture *In Vitro*

For 3D spheroid formation, the hanging-drop method was applied. In order to create a hanging drop with cells, the cover was removed from the tissue culture dish and 5 × 10^5^ CT26 or 4T1 cells were placed at the bottom of the dish in 10 μL drops. In all cases, the homogeneous, single-cell suspensions were applied. The cover was then reversed to the bottom chamber filled with sterile PBS. Next, the prepared cells were cultured at 37°C with 5% CO_2_ under fully humidified conditions. Spheroids growth was monitored, and the culture medium (DMEM with 10% FBS and 1% of antibiotics) was carefully changed every day. Their culture was continued until round-shaped aggregates of cells were formed inside the hanging drops. After this time, spheroids were transferred to 96-well plates precoated with the Geltrex matrix. Spheroids were allowed to grow until they reached the optimal sizes in diameter (assessed by optical microscopy imaging of living spheroids). In general, after 4–7 days, spheroids were found to be optimal for AgNPs testing. For this purpose, the spheroids were incubated with the investigated NPs at a 1 μg/mL concentration for 24 h. After the incubation, spheroids were rinsed twice with prewarmed PBS and stained with Hoechst33342 (15 min), Calcein AM (45 min), and PI (15 min) to assess the live/dead cell population. Then, spheroids were washed two times and visualized under a Zeiss LSM 880 confocal microscope (Carl Zeiss, Jena, Germany) with a 10× objective. Images were captured and analyzed using Zeiss ZEN software.

#### hiPSC Culture and Differentiation to Colonic Organoids

hiPSCs were cultured on Geltrex (Thermo Fisher) precoated 6-well plates in mTeSR1 growth medium (STEMCELL Technologies). Cells were grown in an incubator at 37°C with 5% CO_2_. The iPSC differentiation to colonic organoids as a model of colorectal cancer was performed using the experimental conditions and protocols described in ref (68). This method is based on the modulation of signaling pathways for the progressive generation of (1) DE with CHIR99021 and activin A, (2) hindgut endoderm (HE) using CHIR99021 and FGF4, and subsequently, colonic organoids (CO) through supplementation with CHIR99021 + LDN19318 + EGF and B27. The time needed for the complete differentiation process reached *ca.* 40 days.

#### hiPSC-Derived Organoid Staining

The expression of selected factors: OCT4, SOX17, CDX2, Nanog, FOXA2, and cyclin D1 was evaluated in organoids. In the beginning, the organoids were fixed in PFA (3.8%) for 30 min. Then, the organoids were rinsed with PBS two times, and 0.1% Triton X-100 solution was added for 90 min. After this time, they were rinsed again with PBS, and samples were incubated with 1% BSA + 0.05% Triton X-100 for 3 h. In the next step, the organoids were incubated with primary antibodies for 12 h at 4°C. Afterward, secondary antibodies were added for 3 h. Hoechst33342 was added to samples 10 min before the end of incubation. Then, organoids were washed with PBS three times and were prepared for confocal images.

The viability of organoids was examined by live/dead staining with PI and Calcein AM. The selected organoids (without and with NPs treatment) were washed twice with HBSS. Then, organoids were stained with Hoechst33342, Calcein AM, and PI to assess live and dead cell populations for 1 h. After this time, they were washed with HBSS twice and prepared for visualization. The samples were imaged using a Zeiss LSM 880 confocal microscope (Carl Zeiss, Jena, Germany) with a 40× immersion objective. Images were recorded and analyzed using Zeiss ZEN software.

#### *In Vivo* Studies

All experiments were performed with permission no. 190/2018 from the 2^nd^ Local Institutional Animal Care and Use Committee (IACUC) in Kraków, Poland. Male, 8–10 weeks old BALB/c mice were purchased from AnimaLab. The mice were housed with a 14/10 h light/dark cycle, and they had access to both food and water ad libitum. In order to assess the Ag@TSC2 toxicity toward cancer, mice were randomly divided into one control group and four experimental groups that were injected with NPs (*N* = 5). For this purpose, mice were intraperitoneally injected with AgNPs solution at a dose of 2 mg/kg twice a week. The tumor sizes were monitored each day, and tumor growth kinetics were evaluated. The weight of mice was controlled, and changes in their behavior were assessed each day during the experiment.

#### Statistical Analysis

Throughout the manuscript, the results were expressed as the mean ± SD or the mean ± standard error of the mean (SEM) from at least three independent experiments. The statistical significance of differences and *P*-values were estimated with the GraphPad Prism 5.0 program (GraphPad Software, San Diego, USA). Differences between groups were compared using two-way ANOVA, and a *P*-value <0.05 (maximum) was considered to be significant.

## Supporting Information Available

Spectroscopic characterization of AgNPs, NP characterization with DLS measurement, NP characterization with XPS measurement, SAED patterns of Ag@TMA1 and Ag@TSC2, XRD analysis, stability of AgNPs under biorelevant conditions, AgNPs interaction with DNA, antibacterial effect of AgNPs in *E. coli* and *S. aureus*, and *in vitro* anticancer activity determined by flow cytometry (PDF)

## Abbreviations

**4T1**: murine mammary gland carcinoma
**8701-BC**: breast cancer cells cultured from primary ductal infiltrating carcinoma
**AgNPs**: silver nanoparticles
**APF**: aminophenyl fluorescein
**ATP**: adenosine triphosphate, energy-carrying molecule
**BALB/c**: common, multifunctional laboratory mouse model
**Caco-2**: caucasian colon adenocarcinoma
**CDX2**: caudal type homeobox 2
**CFU**: colony-forming unit
**CT26**: murine colon carcinoma
**DE**: definitive endoderm
**DLS**: dynamic light scattering
**DMEM**: Dulbecco’s Modified Eagle Medium
**DNA**: deoxyribonucleic acid
**EDX**: energy-dispersive X-ray spectroscopy
**FDA**: Food and Drug Administration
**FOXA2**: forkhead box A2
**H1N1**: influenza type A virus, popularly named “swine flu”
**HaCaT**: non-neoplastic human keratinocytes
**HCT116**: cell line isolated from a colon cancer patient
**hiPSC**: human induced pluripotent stem cell
**HT-29**: human colon cancer cell line
**ICP**: inductively coupled plasma
**MCF-7**: Michigan Cancer Foundation-7, breast cancer cell line
**MDR**: multidrug resistance
**MIC**: minimum inhibitory concentration
**NPs**: nanoparticles
**OCT4**: octamer-binding transcription factor 4
**PdI**: polydispersity index
**PI**: propidium iodide
**ROS**: reactive oxygen species
**SAED**: selected area electron diffraction
**SARS-CoV-2**: severe acute respiratory syndrome coronavirus 2
**SEM**: scanning electron microscopy
**SKBR3**: human breast cancer cell line that overexpresses the Her2 (human epidermal growth factor receptor 2)
**SOX17**: SRY-Box transcription factor 17
**TEM**: transmission electron microscopy
**TM**: temperature melting point
**TMA**: *N*,*N*,*N*-trimethyl-(11-mercaptoundecyl) ammonium chloride
**TSC**: trisodium citrate
**XPS**: X-ray photoelectron spectroscopy.
