# Supplementary material for: Synthesis and Characterization of Size- and Charge-Tunable Silver Nanoparticles for Selective Anticancer and Antibacterial Treatment
Source: ACS Appl Mater Interfaces. 2022 Mar 28;14(13):14981–96. doi: 10.1021/acsami.2c01100 (PMC8990520; doi:10.1021/acsami.2c01100)
Supplement: Supplementary file 1 — am2c01100_si_001.pdf [file am2c01100_si_001.pdf]

Supporting information

Synthesis and characterization of size- and charge-tunable silver nanoparticles for selective anticancer and antibacterial treatment

Barbara Pucelik[1], Adam Sułek[2], Mariusz Borkowski[3], Agata Barzowska[1], Marcin Kobielusz[2] and Janusz M. Dąbrowski[2*]*

[1] Małopolska Centre of Biotechnology, Jagiellonian University, 30-387 Kraków, Poland

[2] Faculty of Chemistry, Jagiellonian University, 30-387 Kraków, Poland

[3] Jerzy Haber Institute of Catalysis and Surface Chemistry Polish Academy of Sciences, 30-239 Kraków, Poland

Correspondence should be sent to Prof. Janusz M. Dąbrowski, jdabrows@chemia.uj.edu.pl *and Dr. Barbara Pucelik,* barbara.pucelik@uj.edu.pl

Spectroscopic characterization of AgNPs. The UV–vis spectrum of the nanoparticles suspension exhibit absorption maximum at 450 nm for Ag@TMA2 and at 420 nm for Ag@TSC1. Ag@TMA2, similarly to Ag@TMA1, has a board absorption band. In the case of Ag@TSC1, there are no evident differences between its smaller counterpart in the UV-vis spectrum. Presented spectra were recorded for NPs at a concentration of 1 μg/mL prior to short sonication to avoid particle agglomeration.

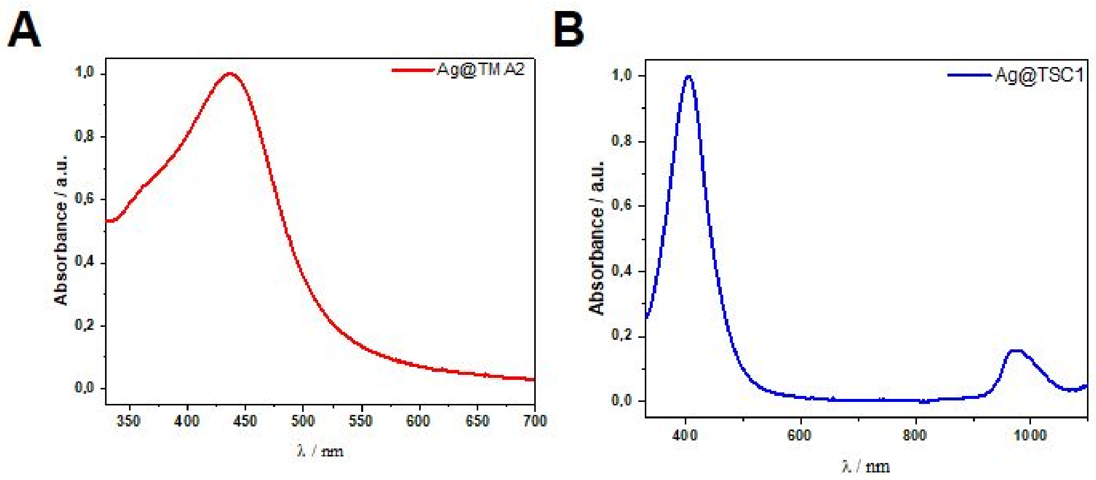

Figure S1. The normalized electronic absorption spectra of Ag@TMA2 (A) and Ag@TSC1 (B).

NPs characterization with DLS measurement.

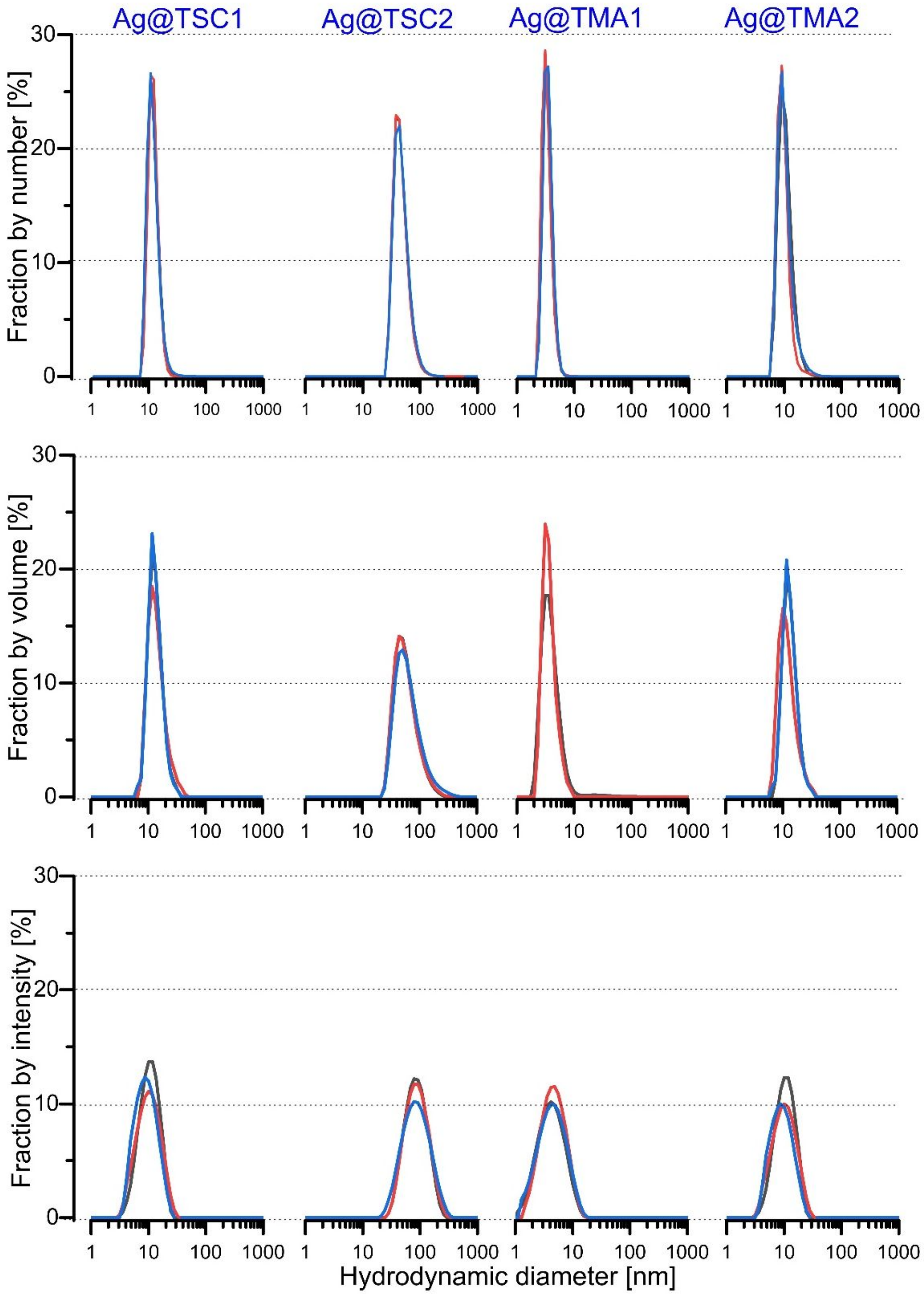

Figure S2. The DLS measurements of tested AgNPs calculated by number, volume, and fraction intensity.

Table S1. DLS analysis for investigated nanoparticles presented by number, volume, and intensity with calculated PdIs, respectively.

Hydrodynamic diameter	Ag@TSC1		Ag@TSC2		Ag@TMA1		Ag@TMA2	
	d ± SD [nm]	PdI	d ± SD [nm]	PdI	d ± SD [nm]	PdI	d ± SD [nm]	PdI
By number	11.5 ±3.4	0.09	41.0±13.7	0.11	3.1±0.6	0.04	9.2±1.2	0.02
By volume	11.8 ±3.3	0.08	49.3±18.3	0.14	3.4±0.9	0.07	11.6±3.3	0.08
By intensity	12.8±4.3	0.11	74.0±37.4	0.26	4.6±2.2	0.23	9.87±4.6	0.21

NPs characterization with XPS measurement. The presence of the attached group has been confirmed using X-ray photoelectron spectroscopy (XPS). XPS spectra were measured in analyzer transmission mode with pass energy 30 eV. Photon energies (PE) of 380 eV were used for C 1*s* and S 2*p* spectral regions, and PE of 596 eV was selected to detect O 1*s*, N 1*s*, and Ag 3*d* core levels spectra, with energy resolution ΔE = 0.22 eV. Spectra analysis was performed with Voigt-shaped peaks and subtraction of a Shirley background. The doublets of S $2p_{3/2}$, S $2p_{1/2}$ and Ag $3d_{5/2}$, Ag $3d_{3/2}$ were fitted using the same full width at half-maximum (FWHM) for each pair of components of the same core level. A spin-orbit splitting of 1.2 eV and 6.0 eV, and branching ratios of 2 and 3/2 were used, respectively.

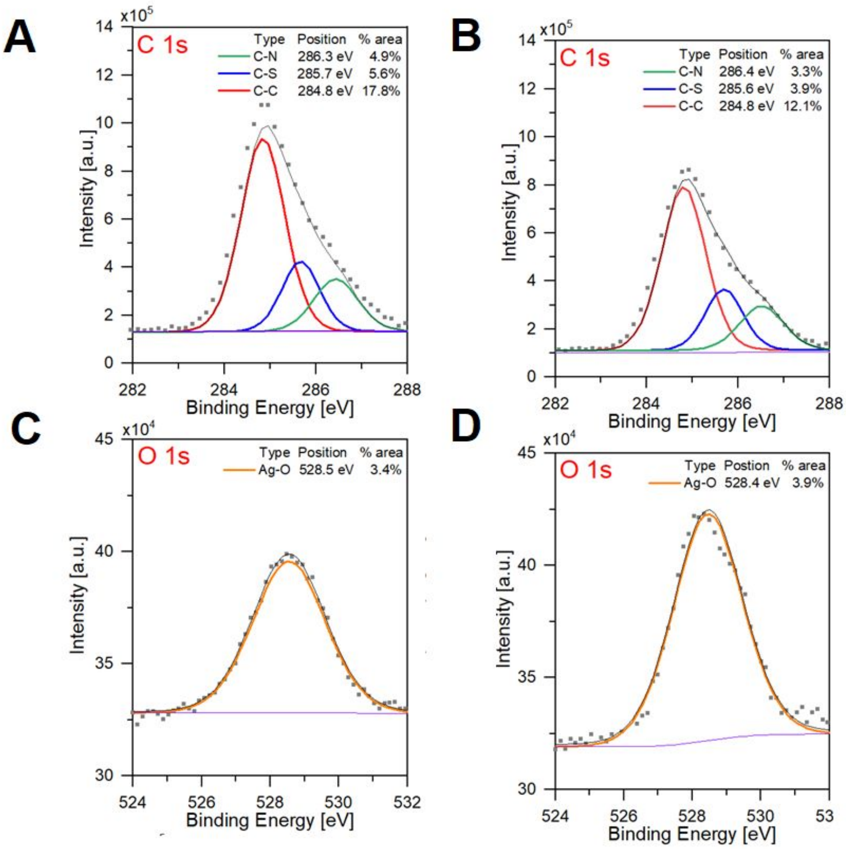

Figure S3. XPS spectra of Ag@TSC1 (A, C) and Ag@TSC2 (B, D) correspond to C and O atoms, respectively. The percentage of components calculated in the CasaXPS program is shown in figure inserts.

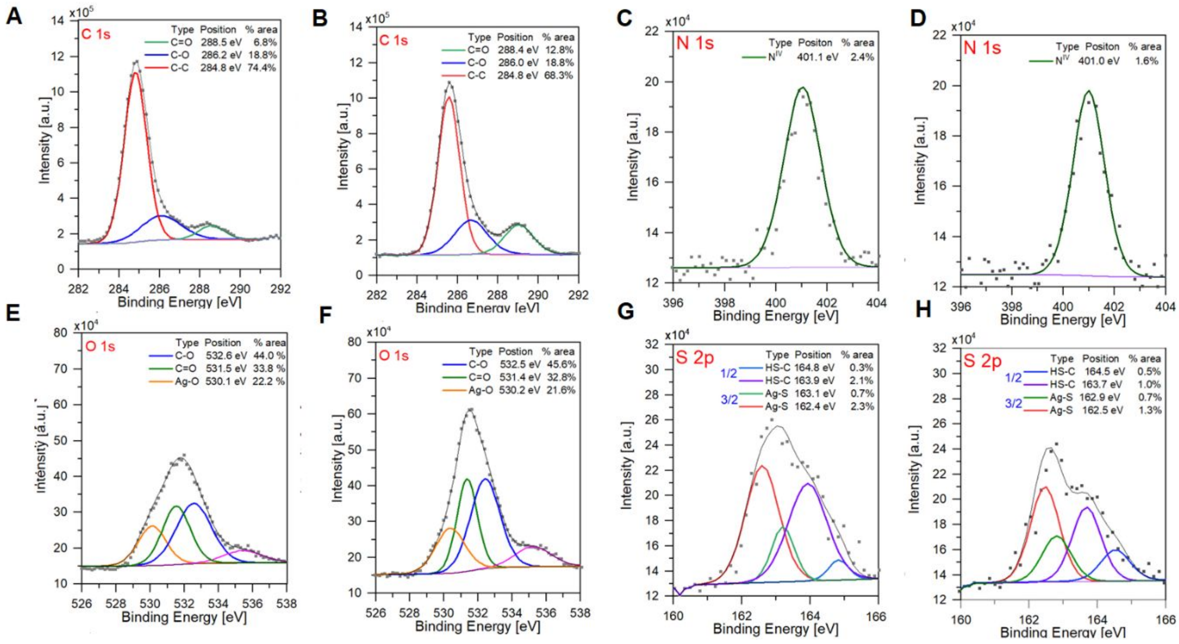

Figure S4. XPS spectra of Ag@ TMA1 (A, C, E, G) and Ag@ TMA2 (B, D, F, H) correspond to C, N, O, and S atoms, respectively.

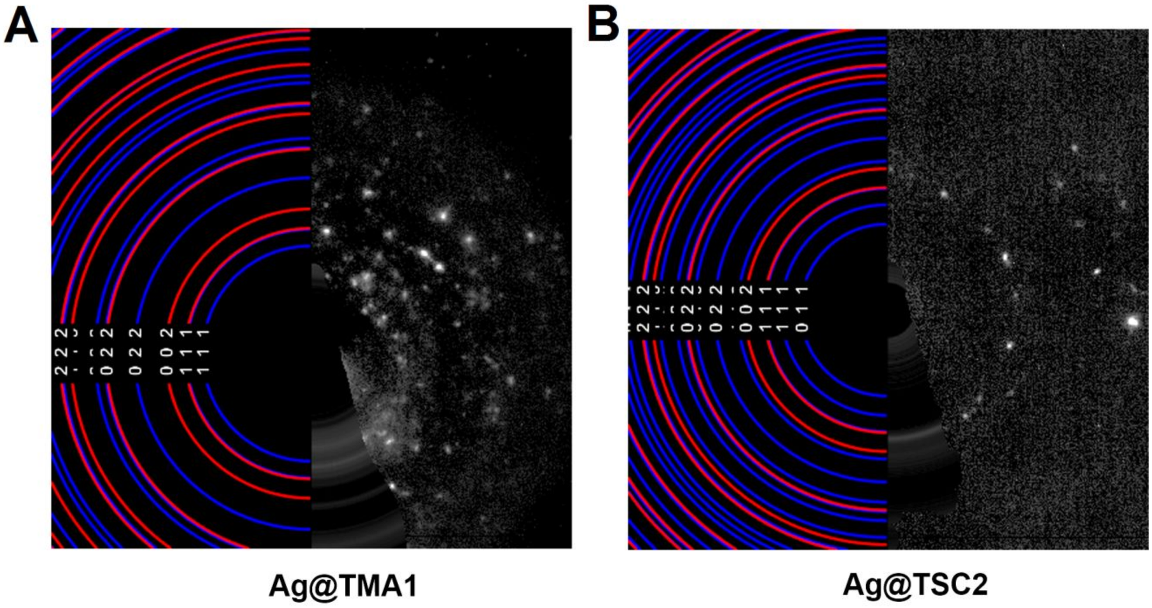

Figure S5. SAED patterns of (A) Ag@TMA1 and (B) Ag@TSC2: red line indicates Ag, blue – AgO, respectively. The fitting of the crystallographic planes to the SAED pattern was made based on M. Klinger and A. Jäger. Crystallographic Tool Box (CrysTBox): automated tools for transmission electron microscopists and crystallographers. *J. Appl. Crystallogr.*, 48(6).

XRD analysis. The crystallinity of the samples was evaluated by powder X-ray diffraction (P-XRD) experiments, measured at room temperature using a Bruker D8 Advance Eco powder X-ray diffractometer equipped with Cu-Kα radiation source and a capillary spinning add-on.

Figure S6. shows the P-XRD patterns of vacuum-dried Ag@TSC1 and Ag@TMA1 samples. Both samples show some well-defined diffraction peaks at *c.a.* 38°, 44° and 64° due to the (111), (200), and (220) reflections of the face-centered cubic metallic silver, respectively. This observation confirms the presence of silver nanoparticles in the tested samples.

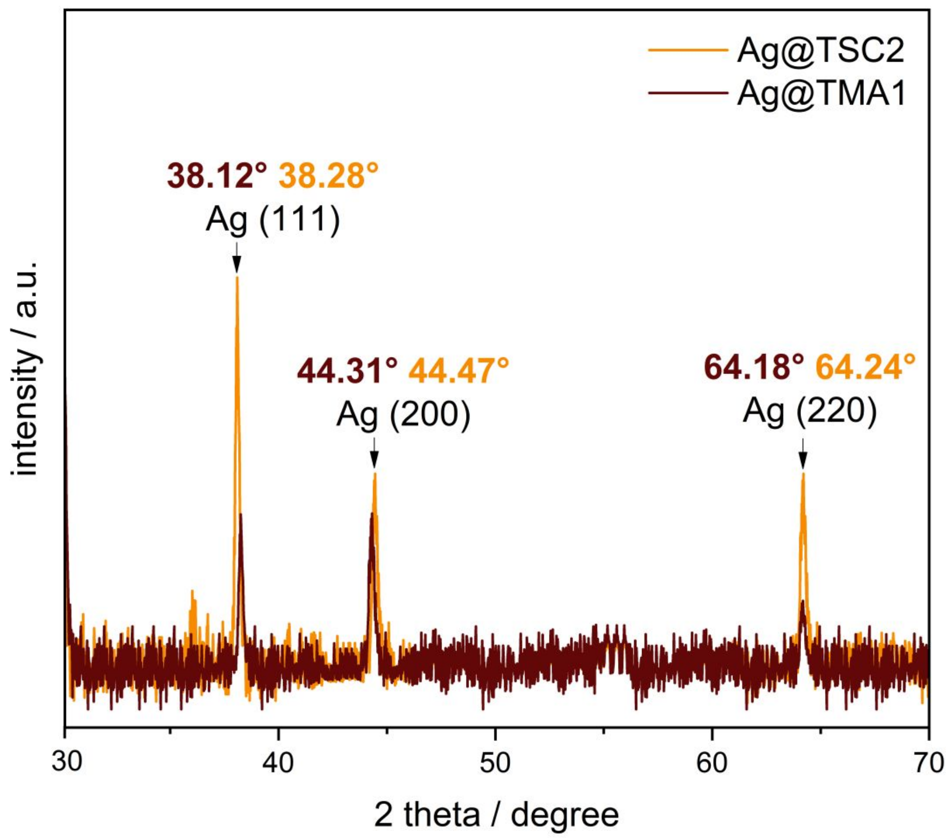

Figure S6. X-ray diffraction patterns of Ag@TSC2 and Ag@TMA1 samples.

Stability of AgNPs in biorelevant conditions. The aggregation behavior of AgNPs is affected mainly by pH and electrolyte concentration, while the presence of biomolecules can improve particle stability due to the biomolecular corona effect. The effects of pH and under biorelevant conditions in DMEM – on the aggregation behavior of these particles in a time-dependent manner to evaluate the nanoparticle aggregation grade and its impact on cytotoxicity. Stability of NPs over 9 days at 37 ^{o}C dispersed at 0.5 µg/mL in DMEM shows stability up to 5 days and then significantly loose absorption band.

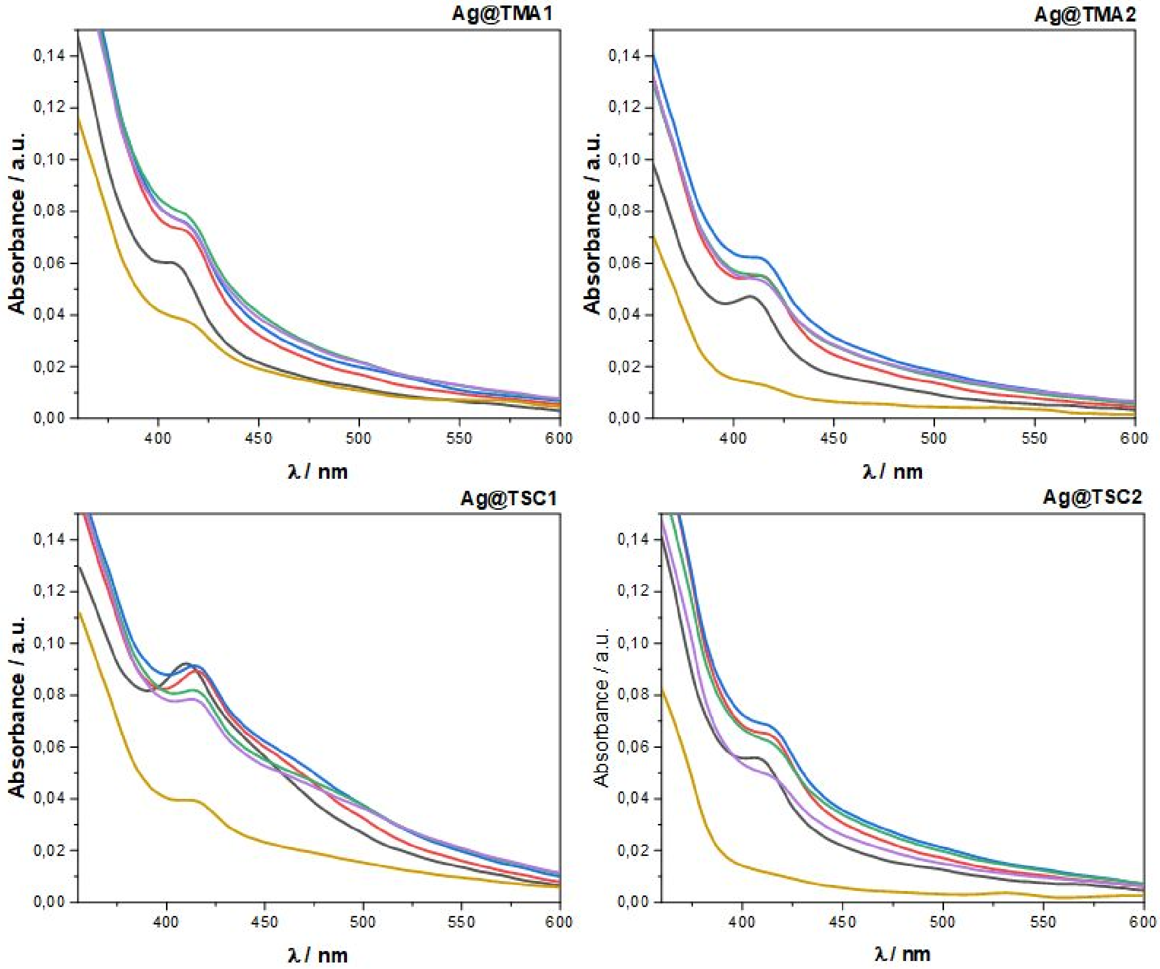

Figure S7. Stability of AgNPs in DMEM cell media monitored by UV-Vis measurements.

AgNPs interaction with DNA. Nanoparticles can interact with DNA covalently as well as non-covalently by intercalation. Depending on the surface functionalization of nanoparticles, charge, and size, there is the possibility to bind in the large or minor groove. Studies on the interaction of NPs and DNA have received great attention due to the possible effects of nanoparticles on the structural integrity of DNA. The experiments were performed by monitoring the absorption of ct-DNA sample at 260 nm as a function of temperature ranging from 20 to 95 °C in the absence and presence of AgNPs. The samples contained either 50 µM ct-DNA alone or 50 µM of ct-DNA and 0.5 µg/mL nM AgNPs each. The volume of the samples was 2 mL.

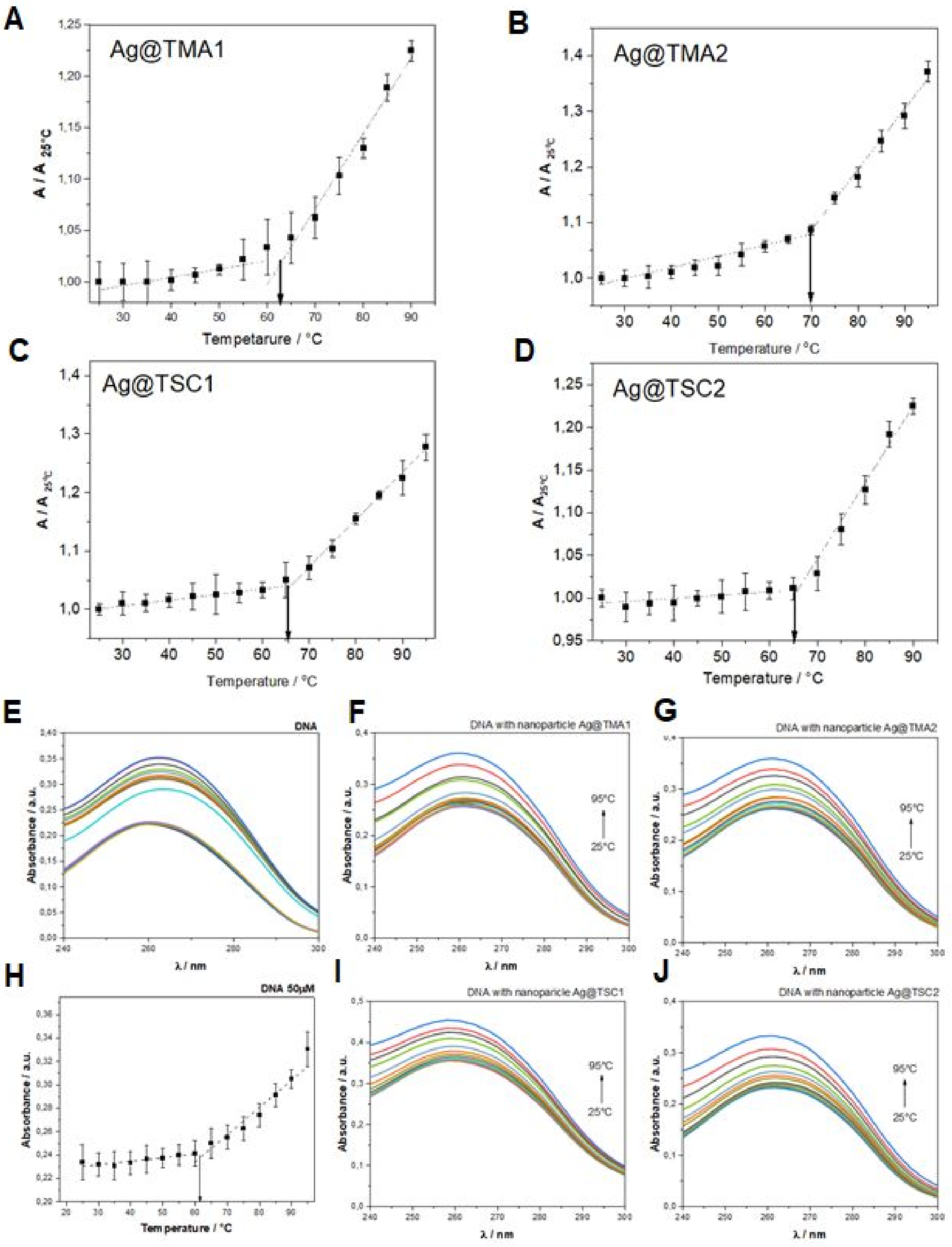

Figure S8. The plot of the absorption intensity of the DNA with continuous melting method (E, H) and in the presence of Ag@TMA1 (A, F), Ag@TMA2 (B, G), Ag@TSC1 (C, I), and Ag@TSC2 (D, J).

Antibacterial effect of AgNPs. To examine the susceptibility of *E. coli* and *S. aureus* to different silver nanoparticles, bacteria were incubated with AgNPs for 2 h. Then, a sample of bacterial suspension was plated on a nutrient agar plate, and the plates were incubated further

at 37°C. The numbers of resultant colonies were counted after 24 h of incubation. Silver-free plates incubated under the same conditions were used as controls.

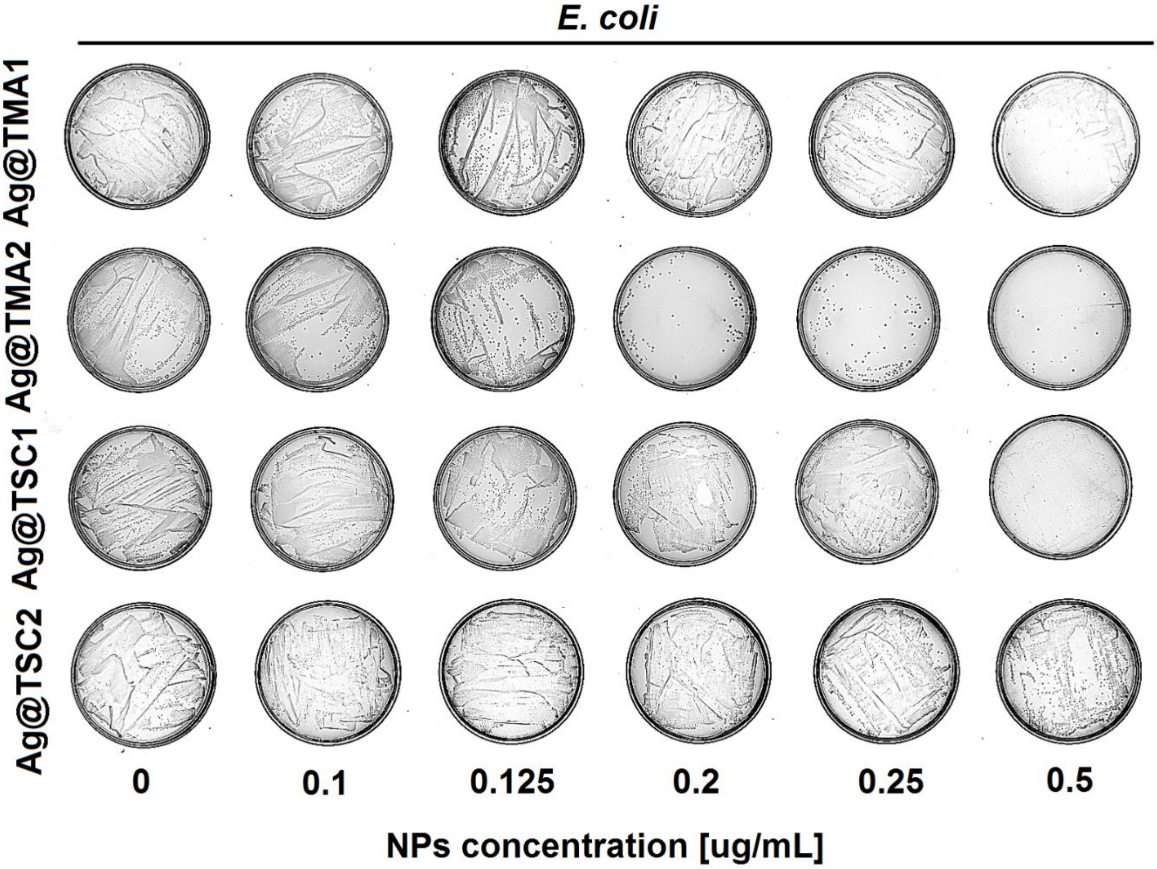

Figure S9. Photographs showing the colonies of *E. coli* in agar Petri dishes after treatment with investigated AgNPs.

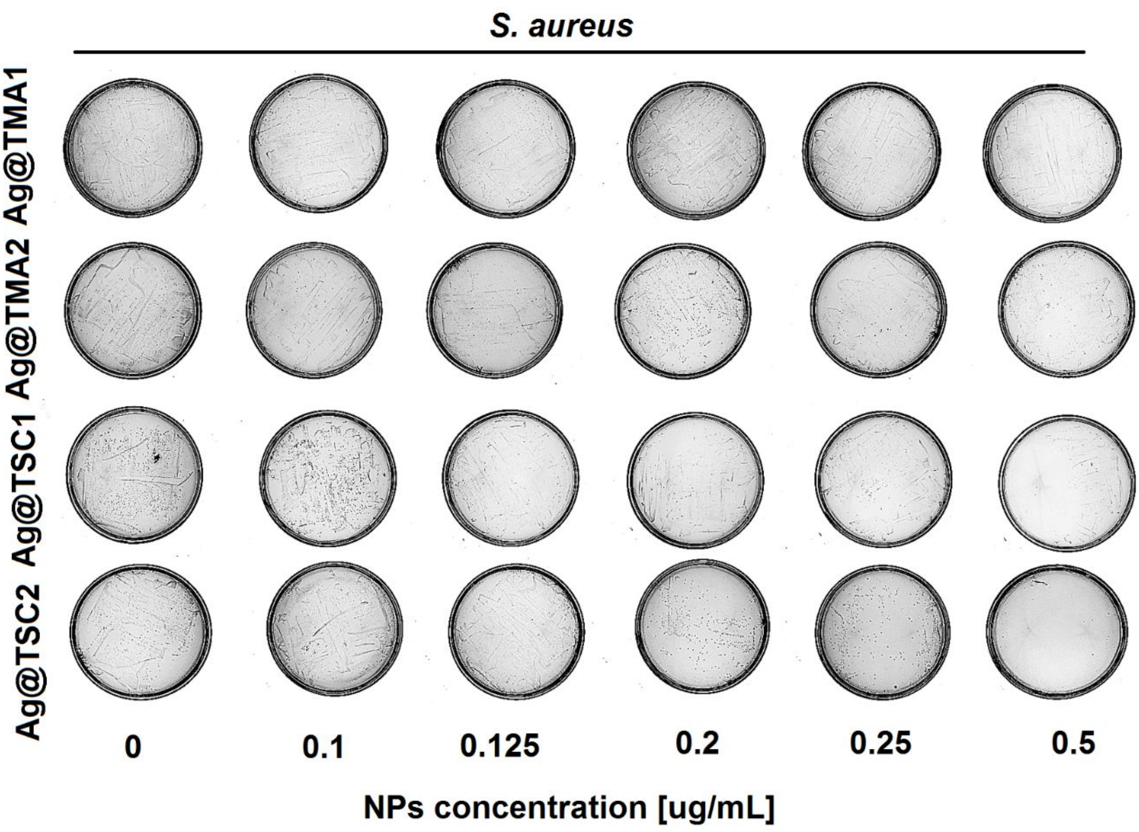

Figure S10. Photographs showing the colonies of *S. aureus* in agar Petri dishes after treatment with investigated AgNPs.

***In vitro* anticancer activity:** Flow cytometry of AgNPs-induced cell death.

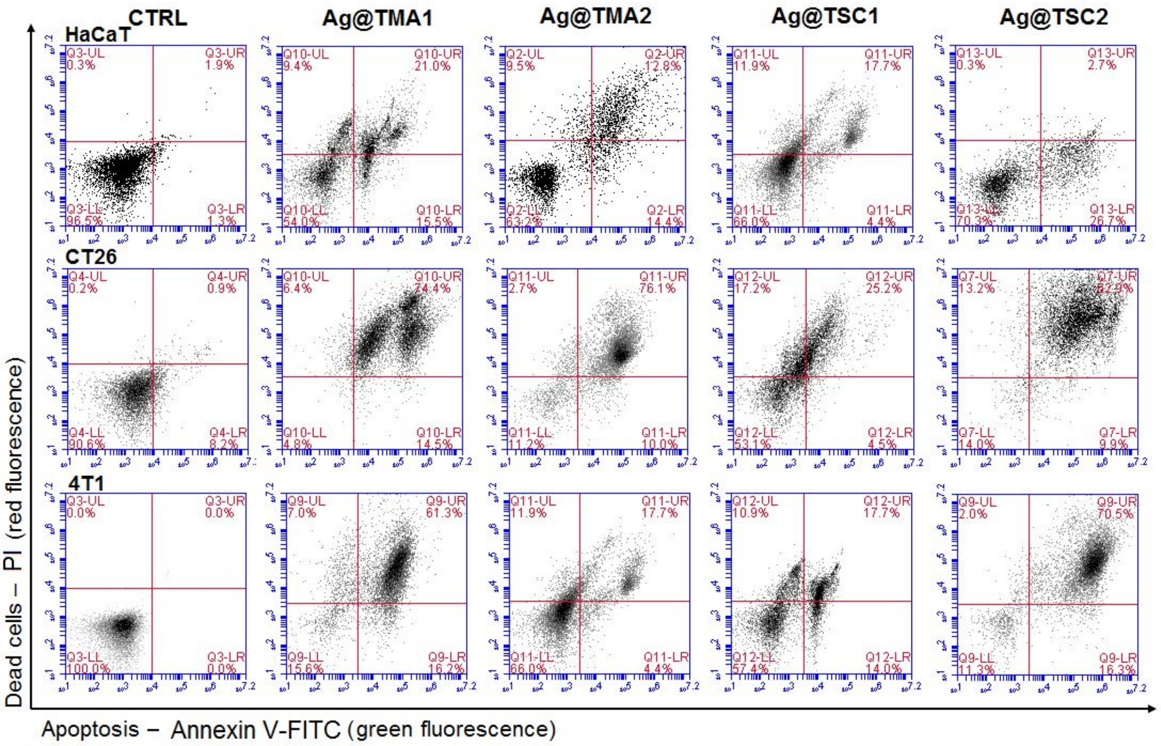

Figure S11. Representative dotplots obtained by flow cytometry showing determination of HaCaT, CT26 and 4T1 cells death modes induced by AgNPs: the Annexin V-FITC/propidium iodide (PI) double staining assay (Annexin V-FITC - green fluorescence, PI - red fluorescence) was used to detectphosphatidylserine externalization in apoptosis and analyse the membrane integrity, respectively.
